# Attosecond three-stage formation and coherent exciton dynamics in a two-dimensional material under strong field

**DOI:** 10.1038/s41377-026-02293-7

**Published:** 2026-04-29

**Authors:** Qing Chen, Daqiang Chen, Chenyu Wang, Yunfei Bai, Chao Lian, Zhe Xu, Haizhong Guo, Enge Wang, Sheng Meng

**Affiliations:** 1 Beijing National Laboratory for Condensed Matter Physics and Institute of Physics, Chinese Academy of Sciences, Beijing, 100190 China; 2 School of Physical Sciences, University of Chinese Academy of Sciences, Beijing, 100190 China; 3 Songshan Lake Materials Laboratory, Dongguan, Guangdong 523808 China; 4 Key Laboratory of Material Physics, Ministry of Education, School of Physics, Zhengzhou University, Zhengzhou, 450001 China; 5 Institute of Quantum Materials and Physics, Henan Academy of Sciences, Zhengzhou, 450046 China

**Keywords:** Optical spectroscopy, Ultrafast photonics

## Abstract

Excitons play a crucial role in optical properties of two-dimensional materials. While significant progress has been made in understanding exciton dynamics on femtosecond timescales, the microscopic details of the earliest stages of exciton formation and evolution remain elusive. Here we explore the ultrafast processes of exciton formation, evolution and dissociation in monolayer hexagonal boron nitride using state-of-the-art time-dependent density functional theory simulations incorporating long-ranged interactions. We find that the exciton forms within ~2.5 fs through a three-step process: free carriers are first generated by photoexcitation; electrons and holes subsequently bind to form a metastable “exciton core”; and finally, the exciton core evolves into a fully-formed exciton. The subsequent dynamics are dominated by exciton-exciton interference, which gives rise to oscillatory electron-occupation signals. These signals serve as a predicted phase-sensitive signature, providing a theoretical basis for experimentally probing the exciton envelope phase. This interference can be further modulated and an anisotropic Mott transition is induced upon increasing laser intensity into the strong field regime.

## Abstract (web summary)

Excitons in h-BN form within ~2.5 fs via a three-step process under resonant excitation.

## Graphical abstract

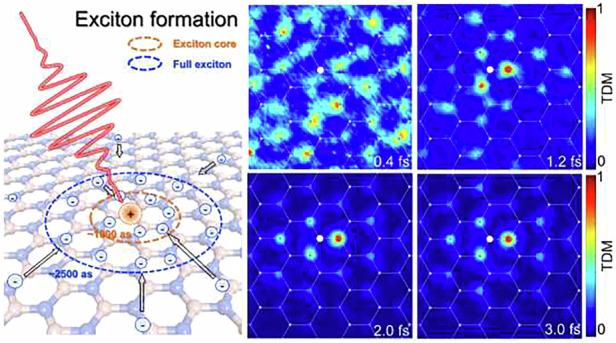

## Introduction

Excitons play a pivotal role in the optical properties of two-dimensional (2D) materials due to their significantly enhanced binding energies and oscillator strengths compared to their counterparts in bulk materials.^1,2^ In particular, the formation of excitons under both resonant and non-resonant excitation has garnered considerable attention,^3–7^ as it is crucial for processes such as light-to-electricity conversion and signal modulation. Understanding exciton dynamics at ultrafast resolutions is essential for advancing both our fundamental knowledge and optoelectronic device applications.^2,8,9^

Significant progress has been made recently in unraveling exciton dynamics. Advanced theoretical simulations have revealed exciton dephasing times,^10–12^ and elucidated the mechanisms by which excitons transfer into free band carriers on femtosecond timescales,^13^ consistent with recent strong-field experimental observations.^14^ Notably, under non-resonant excitation, exciton formation has been shown to occur on timescales of tens of femtoseconds through phonon-mediated relaxation cascades.^6^ By contrast, the formation of excitons under resonant excitation remains far less well understood. Historically, exciton formation in this regime is often reported as “instantaneous”,^5^ largely because quantitative formation times are difficult to extract. Although sub-10-fs temporal resolution is now achievable in ultrafast spectroscopy,^15–17^ the direct observation of exciton formation under resonant excitation remains challenging due to the lack of strong signatures associated with excitonic dynamics. Consequently, microscopic formation pathways and formation times under resonant excitation have remained largely inaccessible. In particular, several fundamental questions remain unanswered: Is this process instantaneous? What are the dynamics after exciton formation? How do band topology,^18^ exciton-exciton interactions,^19^ and external light fields^12,20^ influence these processes?

To address these questions, advanced theoretical approaches are required to accurately capture ultrafast excitonic processes on the sub-femtosecond timescale. In this context, considerable expectations have been placed on real-time nonequilibrium Green’s function theory^21,22^ and real-time time-dependent density functional theory (rt-TDDFT)^23–25^ as promising approaches for investigating the very early stage of exciton dynamics. While time-dependent adiabatic GW approaches significantly advanced our understanding of exciton dynamics,^26,27^ the perturbative nature and the use of the statically screened interaction *W* limit their ability to describe exciton evolution under strong fields. Although a GW-ODE scheme has been proposed recently, enabling *W* to be dynamically updated during time evolution, its application remains restricted to systems with only a few electronic bands.^28^

On the other hand, rt-TDDFT has shown great potential for capturing real-time electron dynamics in both real and momentum space. Conventional functionals adopted in regular rt-TDDFT simulations, such as adiabatic local density approximations^29^ struggle to capture excitonic effects in extended systems, due to the lack of the correct long-range asymptotic behavior.^30,31^ A significant advance has been made by employing range-separated hybrid (RSH) functionals.^32^ By incorporating Fock exact exchange potential in the long range to replace that in local density approximation (LDA), the new approach achieves a correct asymptotic behavior. In particular, linear response TDDFT with the RSH functionals has already been used with surprising success in calculating the absorption spectra of materials with significant exciton effects.^33,34^ However, due to the substantial computational cost associated with evaluating Fock exchange at each step of dynamical evolution, studies that employ rt-TDDFT with the RSH functionals (TD-RSH) for exciton dynamics remain rather limited.

**Fig. 1:**
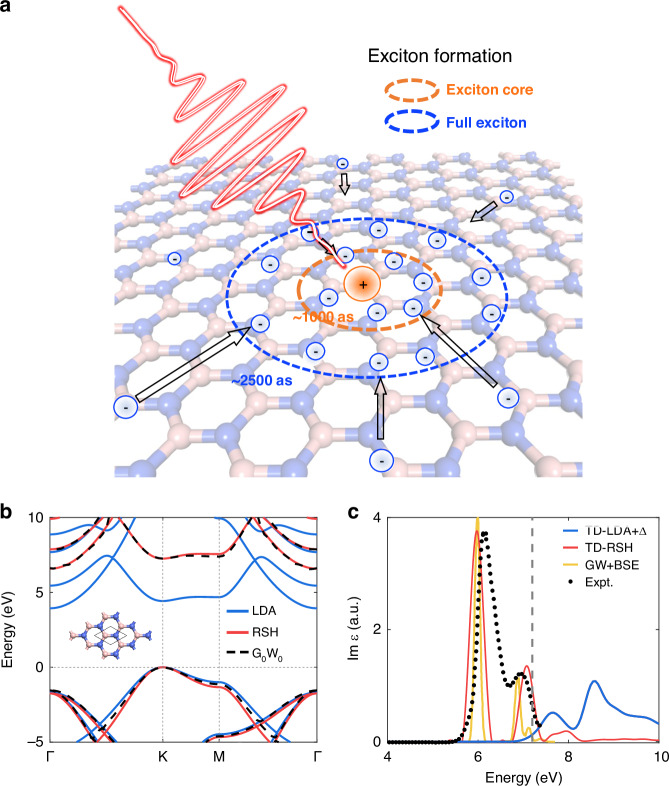
The band structure and the imaginary part of dielectric function of monolayer h-BN. **a** Schematic of the ultrafast exciton formation process. **b** The band structure of monolayer h-BN calculated using DFT with the LDA functional (blue), DFT with the RSH functional (red) and G_0_W_0_ method (black dotted line). The inset depicts the atomic configuration of monolayer h-BN, with boron atoms in pink and nitrogen atoms in blue. **c** The imaginary part of dielectric function of monolayer h-BN calculated using TD-LDA plus a scissor correction Δ = 2.8 eV (blue) and TD-RSH (red). The experimental result^41^ (black dot) and GW + BSE result^57^ (yellow) are shown for comparison

Here, we employ TD-RSH to investigate exciton dynamics in a prototype 2D material, monolayer hexagonal boron nitride (h-BN), well known for its strong excitonic effects.^35^ A series of acceleration algorithms^36^ have been employed in our homemade rt-TDDFT software TDAP,^37–39^ significantly reducing computational costs and making it feasible to simulate exciton dynamics across a wide range of field strengths. Our simulations reveal a three-step exciton formation process occurring on a few-femtosecond timescale in monolayer h-BN, with a representative formation time of ~2.5 fs under resonant excitation (Fig. 1). Furthermore, we demonstrate that exciton-exciton interference plays a dominant role in the post-excitation dynamics, which manifests as oscillatory electron population distributions across different k-points. These oscillations are directly correlated with the phase of the exciton envelope function, providing a potential experimental signature for probing the exciton’s phase information. As the laser field strength further increases, these oscillations diminish and indicate the onset of an anisotropic Mott transition. Our results provide a comprehensive picture of ultrafast exciton dynamics under strong resonant excitation, offering both a deeper understanding of the fundamental mechanisms of exciton formation and theoretical guidance for future experimental studies on excitonic effects.

## Results

### Accurate description of excitons in monolayer h-BN via TD-RSH

The RSH functional separates Coulomb interactions into short-range and long-range components via the error function:

1 $$\frac{1}{r}=\frac{1-\left[\alpha +\beta {\rm{erf}}\left(\gamma r\right)\right]}{r}+\frac{\alpha +\beta {\rm{erf}}\left(\gamma r\right)}{r}$$

where α, β, and γ are tunable parameters. The exchange-correlation energy is then partitioned as:

2 $$\begin{array}{c}{E}_{{\rm{XC}}}=\alpha {E}_{{\rm{HFX}}}^{{\rm{SR}}}+\left(1-\alpha \right){E}_{{\rm{LDAX}}}^{{\rm{SR}}}+\left(\alpha +\beta \right){E}_{{\rm{HFX}}}^{{\rm{LR}}}\\ +\left[1-\left(\alpha +\beta \right)\right]{E}_{{\rm{LDAX}}}^{{\rm{LR}}}+{E}_{{\rm{LDAC}}}\end{array}$$

The parameters are determined through a three-step procedure. First, to ensure the correct asymptotic behavior of the exchange interaction, we impose the condition $\alpha +\beta =1$. Second, the parameters α and γ are further constrained along a one-dimensional curve by fitting to quasiparticle band gaps obtained from first-principles GW calculations. Finally, we set $\alpha =0$, based on the physical consideration that short-range local-field effects in two-dimensional insulators are adequately captured by standard local functionals such as LDA or PBE. This systematic protocol uniquely specifies all parameters, yielding a functional that accurately incorporates both short-range local-field effects and long-range exchange interactions. A detailed discussion of the universality of the parameter determination procedure and the influence of parameters on the results is provided in Supplementary Note S2 of the Supplemental Material.

To explore the dynamics of exciton formation in monolayer h-BN, the ground-state and excitonic properties were first calculated using the RSH functional. In Fig. 1b, we compare the band structure calculated using RSH with results from LDA and G_0_W_0_ methods.^40^ Notably, the RSH functional predicts monolayer h-BN to be an insulator with an indirect gap of 6.58 eV and a secondary direct gap of 7.26 eV located at the K point, consistent with the G_0_W_0_ results (6.62 eV and 7.26 eV), while with LDA functionals the direct gap is severely underestimated at 4.47 eV. More importantly, the absorption spectrum calculated with TD-RSH (Fig. 1c) reveals two prominent below-gap excitations at 5.95 eV and 7.05 eV, corresponding to the 1S and higher-order excitonic states, respectively. The peak positions and intensities are in good agreement with experimental results,^41^ confirming that the RSH functional effectively captures pronounced excitonic effects.

**Fig. 2:**
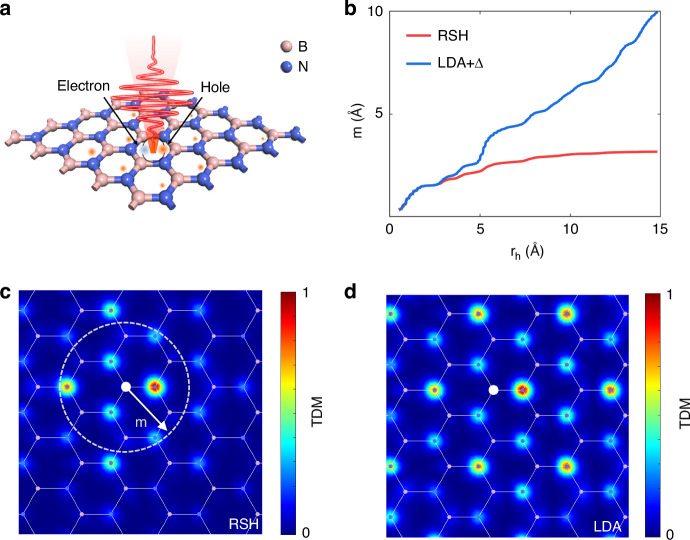
Differences in TDM calculated by LDA and RSH. **a** Schematic diagram illustrating the resonant excitation of monolayer h-BN under laser illumination. **b** The relationship between the exciton radius *m* and the integral radius *r* after laser termination, calculated by TD-RSH (red) and TD-LDA + Δ (blue). **c** and **d** show the TDM $|{\Gamma }_{{\rm{s}}}\left({r}_{{\rm{e}}},{r}_{{\rm{h}}}\right)|$ calculated by TD-RSH and TD-LDA+Δ, respectively, after laser termination (at 10 fs). Here, ${r}_{{\rm{e}}}$ is fixed at the position of a particular boron atom, marked with a white dot. The plotted value of each point has been normalized to its overall largest value in the real space

Besides the absorption spectrum, a detailed examination of the real-space distribution of exciton wavefunctions is necessary to fully understand the nature of exciton binding and localization. Here, we perform resonant excitation of the 1S exciton state using a linearly polarized Gaussian-envelope laser pulse with a duration of 8 fs, as schematically shown in Fig. 2a. The pulse has an energy broadening of ~2 eV, with the electric field reaching its maximum value at 4.2 fs (Supplementary Fig. S8 in the Supplemental Material). After the laser pulse ends, the single-particle transition density matrix (TDM) ${\Gamma }_{{\rm{s}}}$ is calculated to represent the electron-hole wavefunctions:^42^

3 $${\Gamma }_{{\rm{s}}}\left({\boldsymbol{r}},{{\boldsymbol{r}}}^{{\prime} },t\right)=\mathop{\sum }\limits_{i}^{{occ}}\left[{\psi }_{i}\left({\boldsymbol{r}},t\right){\psi }_{i}^{* }\left({{\boldsymbol{r}}}^{{\prime} },t\right)-{\psi }_{i}\left({\boldsymbol{r}},0\right){\psi }_{i}^{* }\left({{\boldsymbol{r}}}^{{\prime} },0\right)\right]$$

where ${\psi }_{i}\left({\boldsymbol{r}},t\right)$ are the Kohn-Sham (KS) orbitals at time *t* in the velocity gauge. By fixing the position of the excited electron on a boron atom (white dot in Fig. 2c, d), the corresponding hole density distribution within the crystal can be visualized.

As shown in Fig. 2c, the TD-RSH simulation reveals a strongly localized hole density around the excited electron, signifying bound excitonic behavior. In contrast, the TD-LDA simulation (Fig. 2d) shows a delocalized hole distribution, consistent with the behavior of free carriers rather than bound excitons. To quantify this difference, the average electron-hole distance, *m*, was evaluated using the formula:

4 $$m=\frac{\int d{r}_{{\rm{h}}}\left|{r}_{{\rm{h}}}\right|{\left|{\Gamma }_{{\rm{s}}}\left({r}_{{\rm{e}}},{r}_{{\rm{h}}},t\right)\right|}^{2}}{\int d{r}_{{\rm{h}}}{\left|{\Gamma }_{{\rm{s}}}\left({r}_{{\rm{e}}},{r}_{{\rm{h}}},t\right)\right|}^{2}}$$

where ${\Gamma }_{{\rm{s}}}$ represents the single-particle TDM, ${r}_{{\rm{e}}}$ is the fixed position of the electron (at a boron atom), and ${r}_{{\rm{h}}}$ is integrated over the spatial distribution of the hole. As shown in Fig. 2b, the TD-RSH result predicts an average electron-hole distance *m* that converges to 3.3 Å, consistent with the bound excitons picture. Conversely, the *m* calculated by TD-LDA fails to converge, as expected for free carriers. These results demonstrate that the TD-RSH overcomes the limitations of TD-LDA in accurately describing electron-hole interactions. By resolving the spatial and energetic characteristics of exciton wavefunctions, TD-RSH establishes itself as a reliable and superior approach for modeling excitonic effects in 2D materials.

### Three-stage exciton formation under resonant excitation

**Fig. 3:**
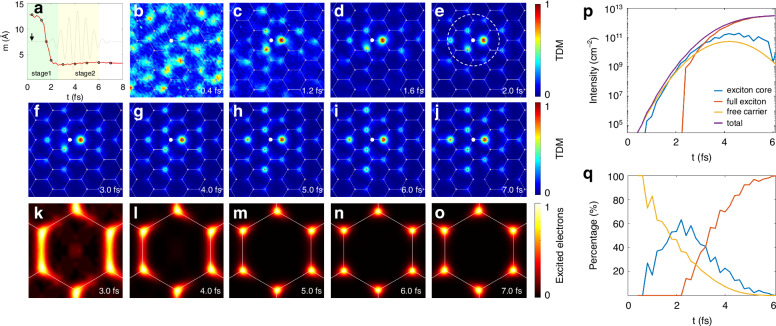
Dynamics of exciton formation in monolayer h-BN. **a** Time evolution of the average electron-hole distance *m*. The laser pulse envelope is depicted by the gray solid line, with the start of the pulse indicated by a black arrow. The green and yellow shaded regions indicate stages of rapid decrease and gradual increase in electron-hole distance, respectively. **b**–**j** The snapshot of TDM $|{\Gamma }_{{\rm{s}}}\left({r}_{{\rm{e}}},{r}_{{\rm{h}}}\right)|$ in real space at the times indicated by black dots in (**a**). The exciton cores are indicated by a dotted white line in figure (**e**). **k**–**o** The snapshot of excited electron distribution in the Brillouin zone (BZ). For both real space and the BZ, values at each point are normalized to their respective maximum intensities. **p** Time evolution of intensity, and **q** proportion of exciton cores (blue), full exciton (red) and free carrier (yellow)

Next, we extend our investigation to the ultrafast dynamics of exciton formation. The monolayer h-BN is excited using the same laser pulse, which is resonant with the 1S exciton state and has an energy broadening of ~2 eV (Supplementary Fig. S8 in the Supplemental Material). Intriguingly, we observe that exciton formation proceeds on a few-femtosecond timescale, within ~2.5 fs under the driving conditions considered here. As shown in Fig. 3a, the average electron-hole distance *m* decreases rapidly from over 10 Å to 3 Å within the first 2.5 fs and then gradually increases to around 3.3 Å by 6 fs. This stepped evolution in *m* suggests distinct behaviors of the photoexcited electron-hole pairs in different stages.

To gain deeper insight into exciton formation, we examine the TDMs at several critical snapshots, as shown in Fig. 3b–j. Initially, at *t* = 0.4 fs the hole distribution is highly delocalized and disordered, indicating that carriers are mainly free particles. Between 0.4 fs and 2.5 fs (Fig. 3c–e), Coulomb attraction drives the hole to localize around nitrogen nearest to the location of the excited electron, forming a bound electron-hole pair. However, this structure differs from the typical 1S exciton, as it lacks the more peripheral parts of the 1S exciton, and hence we name it the “exciton core”, which can be considered a preformed, transient, intermediate state (white dotted circle) during exciton formation. Moreover, we observe an enhanced hole density in regions where the electron-hole polarization is parallel to the pump polarization, indicating that this process is sensitive to external laser field.

After 2.5 fs, the hole density localized around the close-by N atoms increases and the TDM pattern becomes stable after *t* = 6 fs, suggesting that some of the exciton cores transform into fully formed 1S excitons (Fig. 3e–j). This transformation is further supported by the evolution of the excited electrons distribution in the Brillouin zone (Fig. 3k–o). At *t* = 3 fs, excited electrons near the K point, responsible for the formation of the 1S exciton, coexist with electrons excited near the M and Γ points. Between 3 fs and 6 fs, the population of 1S excitons becomes increasingly dominant.

To quantitatively analyze how excitons and free carriers evolve and redistribute over time, we categorize photocarriers into three distinct groups: free carriers, exciton cores, and full excitons. Here, we operationally define an exciton core as the short-range component of the excitonic wavefunction, confined within the average exciton radius. While such a definition does not correspond to a sharply localized entity in real space, it provides a consistent way to monitor the transient conversion between different carrier species. Figure 3p, q show the time evolution of carrier intensities and their relative proportion. It is clear that the rapid reduction in electron-hole separation within the first few femtoseconds corresponds to the formation of exciton cores, followed by an increase in separation as exciton cores transition to fully formed excitons. The delay between the exciton core and full exciton formation can be attributed to the gradual establishment of long-range correlation.

These findings indicate that resonant excitation produces excitons via a rapid three-step process on a few-femtosecond timescale: first, free carriers are generated by photoexcitation; next, these carriers localize under the combined effects of Coulomb attraction and the electric field, forming exciton cores; finally, these exciton cores evolve into fully formed excitons. Additional simulations using weaker resonant fields and different pulse durations (see Supplementary Note S3 in the Supplemental Material) confirm that this three-step mechanism is robust, while the precise timing of the initial localization stage shows a moderate dependence on the pump parameters.

This few-femtosecond formation time is considerably shorter than the ~10 fs reported for non-resonant excitation in monolayer MoS_2_,^6^ consistent with the absence of slower, phonon-mediated relaxation pathways under resonant conditions. The difference might be attributed to the combined absence of phonon-assisted relaxation under resonant excitation and the exceptionally strong intrinsic exciton binding energy in h-BN.

While resolving the three-step exciton formation process directly in real space remains challenging, we predict that the early-stage exciton formation dynamics will be imprinted in the evolution of the carrier energy distribution. The system is initially dominated by free carriers, and as exciton cores form, we anticipate a dynamic spectral weight transfer toward lower energies. Experimentally, this evolution would manifest as a downward shift of the energy centroid of the time-resolved photoemission spectrum. Since this correlation-induced spectral redistribution occurs within a few femtoseconds, it falls, in principle, within the temporal resolution of state-of-the-art ultrafast experiments. However, we note that while similar timescales have been investigated for core-level excitons,^43^ the resonant excitation regime discussed here involves a lower density of excited carriers in the early stages, which may pose challenges in achieving sufficient signal-to-noise for experimental detection.

### Coherent exciton interference in time domain

**Fig. 4:**
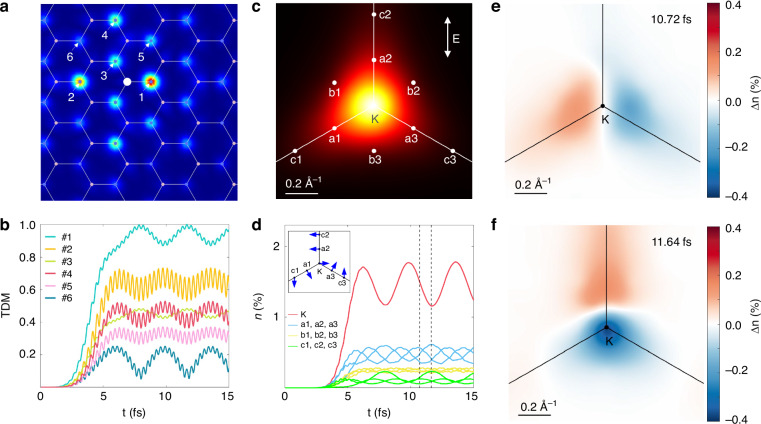
Coherent excitonic beats in monolayer h-BN. **a** Six representative points labeled in real space. **b** Time evolution of the TDM $|{\Gamma }_{{\rm{s}}}\left({r}_{{\rm{e}}},{r}_{{\rm{h}}}\right)|$ at these representative points. **c** Ten representative points labeled around the K point in the BZ. **d** Time evolution of the excited electron population at these representative points. Inset: Phase of the envelope function of the |2P,2S〉 states extracted from the oscillations in electron population. Interference patterns at 10.72 fs (**e**) and 11.64 fs (**f**), with red and blue indicating, respectively, increases and decreases in excited electron population relative to the average at each corresponding time point

We then focus on the dynamics after a stable exciton structure has formed. As depicted in Fig. 4a, b, after 6 fs, the TDM at various sites exhibits periodic oscillations overlaid with a higher-frequency component. This oscillation is also observed in the electron occupation across different k-points (Fig. 4d). Fourier transform analysis (Supplementary Fig. S9b in the Supplemental Material) reveals the high-frequency oscillation at 11.9 eV, corresponding to the second harmonic of the laser pulse. The low-frequency oscillation corresponds to the energy of 1.1 eV, which matches the energy difference of 1.1 eV between the 1S and 2P/2S exciton states. This indicates that the low-frequency oscillation represents a coherent exciton beat, arising from the interference between the excited 1S exciton and 2P/2S excitons.

To further explore the origin of this oscillation, we modeled the dynamical process by assuming that both the 1S and 2P/2S excitons are excited simultaneously and the corresponding exciton states can be expressed as:

5.1 $$\left|1{\rm{S}}\right\rangle =\mathop{\sum }\limits_{{\boldsymbol{k}}{vc}}{A}_{{\boldsymbol{k}}{vc}}\left|{\boldsymbol{k}}{vc}\right\rangle$$

5.2 $$\left|2{\rm{P}},2{\rm{S}}\right\rangle =\mathop{\sum }\limits_{{\boldsymbol{k}}{vc}}{B}_{{\boldsymbol{k}}{vc}}\left|{\boldsymbol{k}}{vc}\right\rangle$$

Here, $|{\boldsymbol{k}}{vc}\rangle$ denotes a free electron-hole pair at the point ***k***, while ${A}_{{\boldsymbol{k}}{vc}}$ and ${B}_{{\boldsymbol{k}}{vc}}$ represent the exciton envelope functions for two excitonic states in reciprocal space, respectively. In the absence of decoherence, the total coherent exciton wave function evolves as:

6 $$\left|{total},t\right\rangle =\left({C}_{1}{A}_{{\boldsymbol{k}}{vc}}{e}^{-i{\omega }_{1}t}+{C}_{2}{B}_{{\boldsymbol{k}}{vc}}{e}^{-i{\omega }_{2}t}\right)\left|{\boldsymbol{k}}{vc}\right\rangle$$

Here, *C*_1_ and *C*_2_ are the amplitudes of the $\left|1{\rm{S}}\right\rangle$ and $\left|2{\rm{P}},2{\rm{S}}\right\rangle$ excitons, and ${\omega }_{1}$ and ${\omega }_{2}$ are their respective oscillation frequencies. The electron occupation at a given k-point can then be expressed as:

7 $${\left|\left\langle {\boldsymbol{k}}{vc}|{total},t\right\rangle \right|}^{2}=\,{\left|{C}_{1}{A}_{{\boldsymbol{k}}{vc}}\right|}^{2}+{\left|{C}_{2}{B}_{{\boldsymbol{k}}{vc}}\right|}^{2}+2|{C}_{1}{C}_{2}{A}_{{\boldsymbol{k}}{vc}}{B}_{{\boldsymbol{k}}{vc}}|cos\left(\left({\omega }_{2}-{\omega }_{1}\right)t+\Delta {\phi }_{{\boldsymbol{k}}}\right)$$

where $\Delta {\phi }_{{\boldsymbol{k}}}$ represents the phase difference between the envelope functions of the two exciton states, defined as: $\Delta {\phi }_{{\boldsymbol{k}}}=\arg ({A}_{{\boldsymbol{k}}{vc}})-\arg ({B}_{{\boldsymbol{k}}{vc}})$. It can be seen from the third term of Eq. (7) that the oscillation in the carrier population contains information about interference between the exciton states. The interference pattern in momentum space at two specific time is shown in Fig. 4e, f, where the distribution features a 2P-like exciton at 10.72 fs along the K-a1 and K-a3 directions, while it can be characterized as a 2S exciton at 11.64 fs along the K-a2 direction. These results further confirm that the observed low-frequency oscillation originates from interference between the 1S and 2P/2S excitons.

Interestingly, because the phase of the envelope function of the 1S state is typically constant over the whole Brillouin zone,^44^ the oscillations of excited electron occupations can, in principle, allow one to retrieve the phase of the exciton envelope function for other excitonic states that interfere with the 1S state. For example, in our simulation, $\Delta {\phi }_{{\boldsymbol{k}}}$ corresponds to the phase of the 2P/2S exciton state envelope function. As shown in the inset of Fig. 4d, the calculated evolution of electron occupation captures the 2π/3 phase shift of the 2P exciton envelope function between points a1 and a2, and the π phase shift of the 2S exciton envelope function at points K and c3.

Previous theoretical studies have shown that coherent exciton beats manifest as temporal oscillations in the momentum-integrated spectrum, with an oscillation period determined by the energy difference between the coherent excitons.^45^ Consequently, the phase shift between exciton beats will correspond to the phase shift in the temporal oscillations of the momentum-integrated spectra along different paths in k-space. While resolving such high-frequency oscillations remains challenging for existing tr-ARPES techniques,^46^ the predicted signatures define a clear experimental target for next-generation implementations in the few-femtosecond regime with enhanced momentum resolution.

Naturally, in a real-world measurement, the effects of dephasing from sources like phonon coupling and exciton-exciton scattering must be considered. In monolayer h-BN, however, the homogeneous linewidth is dominated by radiative recombination (~25 meV), corresponding to a coherence time of ~53 fs.^47^ This timescale is consistent with recent real-time simulations^48^ and ultrafast measurements,^49^ which report comparable decay times in valley carrier dynamics in monolayer h-BN. Our simulations focus on ultrafast dynamics (<20 fs), so the first few oscillations of coherent beats remain largely unaffected. Furthermore, exciton-exciton interactions are naturally included at the mean-field level in the rt-TDDFT framework, while higher-order scattering processes are expected to influence the dynamics only on longer timescales. Therefore, we expect that the predicted phase-dependent oscillations associated with coherent exciton beats could be observable under realistic experimental conditions.

### Field-dependent exciton dynamics

**Fig. 5:**
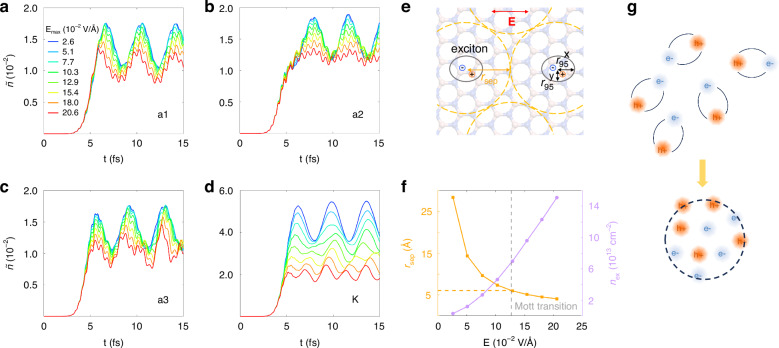
Effect of laser field strength on coherent excitonic beats. Time evolution of the population of excited electrons at points a1 (**a**), a2 (**b**), a3 (**c**), and the K point (**d**). The maximum electric field intensity is represented by a color gradient, with the intensity increasing from blue to red. Each curve is normalized by the total number of input photons. **e** Schematic illustration of the half inter-exciton distance ${r}_{{\rm{sep}}}$ (yellow arrow) and the exciton radii ${r}_{95}$ (black arrows) enclosing 95% of the electron-hole probability density. The red arrow indicates the pump polarization direction. **f** The half inter-exciton distance ${r}_{{\rm{sep}}}$ (left axis) and exciton surface density *n*_ex_ (right axis) as functions of the external excitation field strength. **g** A schematic illustration of exciton dissociation and the formation of electron-hole liquids

Finally, we explore the field dependence of exciton dynamics under resonant pumping conditions. As shown in Fig. 5a–d, all excitation processes exhibit behavior consistent with Pauli blocking, evidenced by a systematic reduction in the average number of excited electrons per photon (denoted $\widetilde{n}$) with increasing electric field intensity in the strong-field regime. Specifically, the coherent excitonic beats exhibit a selective suppression. The beat amplitude at a2 and K diminishes markedly with increasing field (Fig. 5b, d), whereas the beats at a1 and a3 remain robust (Fig. 5a, c).

The selective suppression observed in Fig. 5a–d suggests that the stability of excitonic coherence may be influenced not only by excitation density, but also by the spatial anisotropy of the exciton. To quantify this effect, we compare the inter-exciton separation with the direction-dependent exciton size in real space. The half inter-exciton distance ${r}_{{\rm{sep}}}$ is defined assuming a uniform exciton distribution in the two-dimensional plane (Fig. 5e). Under this assumption, the mean inter-exciton distance is approximated as ${d}_{{\rm{ex}}}=\frac{1}{\sqrt{{n}_{{\rm{ex}}}}}$, where ${n}_{{\rm{ex}}}$ denotes the exciton surface density. Accordingly,

8 $${r}_{{\rm{sep}}}\equiv \frac{{d}_{{\rm{ex}}}}{2}=\frac{1}{2\sqrt{{n}_{{\rm{ex}}}}}$$

This quantity provides a characteristic length scale associated with the effective spatial extent available to a single exciton. When it becomes comparable to the exciton size, significant wavefunction overlap between neighboring excitons is expected.

The exciton size is characterized by a direction-dependent 95% cumulative electron-hole separation radius, which captures the anisotropic real-space exciton distribution induced by the external field. The x-axis is chosen to be parallel to the pump polarization direction. For a given Cartesian direction $\alpha =x,y$, the marginal probability density is defined as

9 $${P}_{x}\left(x\right)={\int }_{-\infty }^{+\infty }{dy}{\left|\varphi \left(x,y\right)\right|}^{2},\,{\,P}_{y}\left(y\right)={\int }_{-\infty }^{+\infty }{dx}{\left|\varphi \left(x,y\right)\right|}^{2}$$

and the cumulative radius ${r}_{95}^{\alpha }$ is determined by

10 $${\int }_{-{r}_{95}^{\alpha }}^{+{r}_{95}^{\alpha }}d\alpha {P}_{\alpha }\left(\alpha \right)=0.95{\int }_{-\infty }^{+\infty }d\alpha {P}_{\alpha }\left(\alpha \right),\,{for}\,\alpha =x,y$$

Here, $\varphi \left(x,y\right)$ denotes the exciton wavefunction extracted at the end of the pump pulse.

As the field increases from 0.026 V·Å^−1^ to 0.206 V·Å^−1^, the half inter-exciton distance ${r}_{{\rm{sep}}}$ decreases from 28.4 Å to 4.1 Å (Fig. 5f). Over the same field range, the cumulative radius ${r}_{95}^{x}$ increases from 5.9 Å to 6.1 Å, whereas ${r}_{95}^{y}$ decreases from 5.2 Å to 4.7 Å. At ~0.127 V·Å^−1^, ${r}_{{\rm{sep}}}$ becomes comparable to the cumulative radius ${r}_{95}$ in the x direction (corresponding to the K-a2 direction in the Brillouin zone). This condition is consistent with the Mott criterion, where enhanced wavefunction overlap and Coulomb screening destabilize excitonic coherence. In contrast, along the direction perpendicular to the electric field, the smaller exciton radius implies that the corresponding stability threshold is reached only at higher fields, leading to a more robust coherence. While strong fields can in principle induce partial exciton dissociation by periodically separating electrons and holes,^12,50^ the persistence of coherent beats in other momentum directions and the weak field dependence of the beat frequencies (Supplementary Fig. S10 in the Supplemental Material) indicate that dissociation alone is unlikely to account for the observed anisotropic suppression of the coherent excitonic beats.

Furthermore, when the maximum field strength exceeds 0.129 V·Å^−1^, a new oscillation frequency at 1.76 eV appears (Supplementary Fig. S10). This frequency increases with carrier density, suggesting that it may originate from plasmons or electron-hole liquids generated by strong field excitation. It should be emphasized that the formation of exciton fluids is generally understood to require stringent conditions, including sufficiently strong dipole-dipole interactions and long coherence times. Although the present results do not directly validate these criteria, the observed density-dependent frequency shift provides a suggestive indication of emergent many-body dynamics, motivating further investigation beyond the scope of this work.

## Discussion

In conclusion, our TD-RSH simulations offer a direct real-time picture of early-stage exciton dynamics in monolayer h-BN, allowing for the explicit tracking of exciton formation, evolution, and dissociation. We demonstrate that exciton formation proceeds via an ultrafast three-step mechanism on a few-femtosecond timescale. The characteristic formation time is ~2.5 fs under resonant excitation. Furthermore, we propose that the electron occupation oscillation arising from coherent exciton beats could serve as a signature for probing the phase of exciton envelope functions. Notably, the disappearance of this oscillatory signal at higher field strengths indicates the onset of the Mott phase transition.

The persistence of these states after photoexcitation and the dynamic interplay between exciton formation and dissociation suggest that excitons in 2D materials can be manipulated in ways that have yet to be fully explored. These findings not only deepen our understanding of exciton dynamics but also open the door to new possibilities in ultrafast optoelectronic applications.

## Methods

### Electronic structure computation

We established an h-BN unit cell model with lattice constants a = b = 2.504 Å, and a vacuum layer of c = 30 Å was used in the calculations to isolate the interactions between layers. The band structure at the DFT-LDA level was calculated using SIESTA software^51^ and at the DFT-RSH level was calculated using HONPAS software,^52,53^ which is based on SIESTA and has computational hybrid functional capabilities. To maintain consistency and ensure convergence in the RSH functional calculations, a double-polarized basis set was used for the B atom, while a double basis set was applied for the N atom in all calculations. Additionally, 18 pseudo-orbitals were added above and below the monolayer h-BN to ensure the completeness of the basis set. A 9 × 9 × 1 auxiliary supercell was utilized to achieve convergence in the RSH functional calculations. An auxiliary real-space grid equivalent to a plane-wave cutoff of 400 Ry was adopted and an 18 × 18 × 1 k-mesh was applied to sample the BZ. The electron-nuclear interactions are described by Norm-Conserving pseudopotentials.^54^

The band structure at the G_0_W_0_ level was calculated using QUANTUM ESPRESSO (QE)^55^ and YAMBO.^56^ In the QE calculations, the DFT ground state was obtained using LDA norm-conserving pseudopotentials with a kinetic energy cut-off at 160 Ry and a 36 × 36 × 1 k-point grid. The G_0_W_0_ calculations were based on the plasmon pole approximation as implemented in the Yambo package, which reads the band structures and wave functions of ground state calculated in QE. We used a truncated Coulomb potential to eliminate the interactions between layers. Moreover, we used a 7 Ry response block size in the dielectric matrix and 200 empty states, which produced the G_0_W_0_ gaps within an accuracy of ≈0.05 eV.

### rt-TDDFT dynamics simulations

The rt-TDDFT simulations were performed using the time-dependent ab initio package (TDAP) as implemented in SIESTA (Algorithm implementation details can be found in the Supplementary Information). The parameters used in the initial state and dynamics calculation are the same as those used in band structure calculation. During dynamic simulations, the evolving time step was set to 0.8 attoseconds for electrons. Since the time scale we focused on is much smaller than the phonon vibration period, we fixed the ion positions in the calculations. The linearly polarized laser pulse was applied to the monolayer h-BN with the waveform

$$E\left(t\right)={E}_{0}\cos \left(\omega t\right)\exp \left[-\frac{{\left(t-{t}_{0}\right)}^{2}}{2{\sigma }^{2}}\right]$$

Here, the photon energy resonates with the 1S exciton state, the half-width of Gaussian wave packet $\sigma$ is 1 fs. In the investigation of exciton generation and coherent exciton beats, the electric field reaches its peak amplitude *E*_0_ = 0.026 V·Å^−1^ at time *t*_0_ = 4.2 fs. For the study of exciton dissociation, the electric field strength is adjusted from 0.026 V·Å^−1^ to 0.206 V·Å^−1^, corresponding to pump intensities of 9.0 × 10^9 ^W·cm^−2^ to 5.6 × 10^11 ^W·cm^−2^.

## Supplementary information

Supplementary Information for Attosecond three-stage formation and coherent exciton dynamics in a two-dimensional material under strong field

## Data Availability

The main data and computational methods supporting the findings of this study are available within the main text and Supplementary Information. The program TDAP is available upon request via http://everest.iphy.ac.cn/tdap and additional data are available from the corresponding authors upon request.
