# Supplementary material for: Attosecond three-stage formation and coherent exciton dynamics in a two-dimensional material under strong field
Source: Light Sci Appl. 2026 Apr 29;15:217. doi: 10.1038/s41377-026-02293-7 (PMC13128943; doi:10.1038/s41377-026-02293-7)
Supplement: Supplementary file 1 — Supplementary Information for Attosecond three-stage formation and coherent exciton dynamics in a two-dimensional material under strong field [file 41377_2026_2293_MOESM1_ESM.docx]

**Supplementary Information for**

Attosecond three-stage formation and coherent exciton dynamics in a two-dimensional material under strong field

Qing Chen^1,2†^, Daqiang Chen^1†^, Chenyu Wang^1,2†^, Yunfei Bai^1,2^, Chao Lian^1,3^*,

Zhe Xu^4^, Haizhong Guo^4,5^*, Enge Wang^1,2,3^*, Sheng Meng^1,2,3^*

^1^Beijing National Laboratory for Condensed Matter Physics and Institute of Physics, Chinese Academy of Sciences, Beijing 100190, China.

^2^School of Physical Sciences, University of Chinese Academy of Sciences, Beijing 100190, China.

^3^Songshan Lake Materials Laboratory, Dongguan, Guangdong 523808, China.

^4^Key Laboratory of Material Physics, Ministry of Education, School of Physics, Zhengzhou University, Zhengzhou 450001, China.

^5^Institute of Quantum Materials and Physics, Henan Academy of Sciences, Zhengzhou 450046, China.

*Corresponding authors. Email: chaolian@iphy.ac.cn; hguo@zzu.edu.cn; egwang@iphy.ac.cn; smeng@iphy.ac.cn

^†^These authors contributed equally to this work.

**Note S1. Time-dependent density functional theory simulations**

The evolution of the system is governed by the time-dependent many-body Schrödinger equation:

$$\begin{aligned} i\hbar\frac{\partial}{\partial t}\Psi\left( \left\{ \boldsymbol{r}_{j} \right\},\left\{ \boldsymbol{R}_{J} \right\},t \right)=H_{\mathrm{tot}}\left( \left\{ \boldsymbol{r}_{j} \right\},\left\{ \boldsymbol{R}_{J} \right\},t \right)\Psi\left( \left\{ \boldsymbol{r}_{j} \right\},\left\{ \boldsymbol{R}_{J} \right\},t \right) \#\left( S1 \right) \end{aligned}$$

Here $\boldsymbol{r}_{j}$ and $\boldsymbol{R}_{J}$ are the positions of the *j*th electron and *J*th ion, respectively. $H_{\mathrm{tot}}$ is the time-dependent Hamiltonian of the system:

$$\begin{aligned} H_{\mathrm{tot}}=\sum_{j} \frac{\hbar^{2}}{2m}\nabla_{j}^{2}+\sum_{J} \frac{\hbar^{2}}{2M_{J}}\nabla_{J}^{2}+V\left( \left\{ \boldsymbol{r}_{j} \right\},\left\{ \boldsymbol{R}_{J} \right\},t \right)+U_{\mathrm{ext}}\left( \left\{ \boldsymbol{r}_{j} \right\},\left\{ \boldsymbol{R}_{J} \right\},t \right) \#\left( S2 \right) \end{aligned}$$

where the potential energy is given by:

$$\begin{aligned} V\left( \left\{ \boldsymbol{r}_{j} \right\},\left\{ \boldsymbol{R}_{J} \right\},t \right)=\sum_{i<j} \frac{e^{2}}{\left| \boldsymbol{r}_{i}-\boldsymbol{r}_{j} \right|}+\sum_{I<J} \frac{Z_{I}Z_{J}}{\left| \boldsymbol{R}_{I}-\boldsymbol{R}_{J} \right|}-\sum_{j,J} \frac{eZ_{J}}{\left| \boldsymbol{r}_{j}-\boldsymbol{R}_{J} \right|} \#\left( S3 \right) \end{aligned}$$

The first two terms in Eq. (S2) represent the kinetic energies of electrons and ions, respectively, while $U_{\mathrm{ext}}$ accounts for the external potential. Since the timescale under consideration is much shorter than the typical phonon oscillation period, the ions can be treated as stationary, allowing us to focus exclusively on the electronic evolution.

By applying the Runge-Gross theorem (*1*), the many-body Schrödinger equation for electrons reduces to the time-dependent Kohn-Sham (TDKS) equation:

$$\begin{aligned} i\hbar\frac{\partial}{\partial t}\psi_{i}\left( \boldsymbol{r},t \right)=\hat{H}_{\mathrm{KS}}\psi_{i}\left( \boldsymbol{r},t \right) \#\left( S4 \right) \end{aligned}$$

where $\psi_{i}\left( \boldsymbol{r},t \right)$ are the Kohn-Sham (KS) orbitals and $\hat{H}_{\mathrm{KS}}$ is Kohn-Sham single-particle Hamiltonian. To simulate the system's response to a time-varying uniform electric field $\boldsymbol{E}\left( t \right)$, the velocity gauge is adopted to preserve the Hamiltonian's translational symmetry. In this gauge, the electric field $\boldsymbol{E}\left( t \right)$ gives rise to a vector potential $\boldsymbol{A}\left( t \right)$:

$$\begin{aligned} \boldsymbol{A}\left( t \right)=-c \int^{t} \boldsymbol{E}\left( t^{'} \right)dt^{'}\boldsymbol{\#}\left( S5 \right) \end{aligned}$$

The KS orbitals in the velocity gauge acquire a phase factor, given by:

$$\begin{aligned} \tilde{\psi}_{i}\left( \boldsymbol{r},t \right)=\exp\left[ -\frac{ie}{\hbar c}\boldsymbol{A}\left( t \right)\cdot\boldsymbol{r} \right]\psi_{i}\left( \boldsymbol{r},t \right) \#\left( S6 \right) \end{aligned}$$

Substituting this transformation into the TDKS equation leads to the velocity-gauge TDKS equations:

$$i\hbar\frac{\partial}{\partial t}\tilde{\psi}_{i}\left( \boldsymbol{r},t \right)=\{\frac{1}{2m}\left[ \hat{\boldsymbol{p}}+\frac{e}{c}\boldsymbol{A}\left( t \right) \right]^{2}+\int d\boldsymbol{r}^{\boldsymbol{'}}\frac{e^{2}}{\left| \boldsymbol{r}-\boldsymbol{r}^{\boldsymbol{'}} \right|}n\left( \boldsymbol{r}^{\boldsymbol{'}},t \right)+{\hat{\tilde{V}}}_{\mathrm{ion}}$$

$+V_{\mathrm{xc}}\left[ \rho\left( \boldsymbol{r,r}^{\boldsymbol{'}},t \right) \right]\}\tilde{\psi}_{i}\left( \boldsymbol{r},t \right)$ (S7)

where ${\hat{\tilde{V}}}_{\mathrm{ion}}$ represents the non-local pseudopotentials in the velocity gauge:

$$\begin{aligned} {\hat{\tilde{V}}}_{\mathrm{ion}}={\exp\left[ -\frac{ie}{\hbar c}\boldsymbol{A}\left( t \right)\cdot\boldsymbol{r} \right]\hat{V}}_{\mathrm{ion}}\exp\left[ \frac{ie}{\hbar c}\boldsymbol{A}\left( t \right)\cdot\boldsymbol{r} \right] \#\left( S8 \right) \end{aligned}$$

Since the exchange-correlation potential $V_{\mathrm{xc}}$ is derived using the RSH functional which includes the Fock exchange term, it depends on the instantaneous single-particle density matrix:

$$\begin{aligned} \rho\left( \boldsymbol{r,r}^{\boldsymbol{'}},t \right)= \sum_{i}^{occ} \tilde{\psi}_{i}\left( \boldsymbol{r},t \right)\tilde{\psi}_{i}^{*}\left( \boldsymbol{r'},t \right) \#\left( S9 \right) \end{aligned}$$

When $\boldsymbol{r=r}^{\boldsymbol{'}}$, the instantaneous single particle density matrix is transformed into an instantaneous density matrix $n\left( \boldsymbol{r},t \right)$.

To compute the absorption spectrum that incorporates excitonic effects, we apply an impulsive field $\boldsymbol{A}(t)=A_{0}\theta(t)$ along the B-N bond direction, where $\theta(t)$ is the Heaviside step function, and subsequently calculate the macroscopic current $\boldsymbol{j}\left( t \right)$:

$$\begin{aligned} \boldsymbol{j}\left( t \right)=\frac{1}{\Omega}\sum_{i}^{occ} \frac{e}{2m}\int_{\Omega} dr\left[ \tilde{\psi}_{i}^{*}\left( \boldsymbol{r},t \right)\hat{\boldsymbol{\pi}}\tilde{\psi}_{i}\left( \boldsymbol{r},t \right)+c.c \right]\boldsymbol{\#}\left( S10 \right) \end{aligned}$$

$$\begin{aligned} \hat{\boldsymbol{\pi}}\boldsymbol{=}\frac{m}{i\hbar}\left[ \boldsymbol{r,}\hat{H}_{KS} \right]\boldsymbol{=-}i\hbar\nabla+\frac{e}{c}\boldsymbol{A}\left( t \right)+\frac{m}{i\hbar}\left[ \boldsymbol{r,}{\hat{\tilde{V}}}_{\mathrm{ion}} \right]\boldsymbol{\#}\left( S11 \right) \end{aligned}$$

Here, $\Omega$ is volume of the cell and $\hat{\boldsymbol{\pi}}$ denotes the generalized momentum operator. By performing a Fourier transform on the macroscopic current, the frequency-dependent conductivity $\sigma(\omega)$ can be obtained, which in turn leads to the derivation of the dielectric function $\varepsilon(\omega)$:

$$\begin{aligned} \sigma\left( \omega\right)=-\frac{c}{A_{0}}\int^{T} {dte}^{i\omega t}f\left( t \right)j\left( t \right)\#\left( S12 \right) \end{aligned}$$

$$\begin{aligned} \varepsilon\left( \omega\right)= 1+\frac{4\pi i\sigma\left( \omega\right)}{\omega} \#\left( S13 \right) \end{aligned}$$

In Eq. S12, $f\left( t \right)$ is a filtering function that prevents a sudden cutoff of the integrand at the end of the time-evolution period 𝑇.

One key tool for probing excitonic effects is the time-dependent transition density matrix (TDM). In the velocity gauge, the TDM is characterized by the following expression:

$$\begin{aligned} \Gamma_{s}\left( \boldsymbol{r},\boldsymbol{r}^{'},t \right)=\sum_{i}^{occ} \left[ \tilde{\psi}_{i}\left( \boldsymbol{r},t \right)\tilde{\psi}_{i}^{*}\left( \boldsymbol{r}^{'},t \right)-\tilde{\psi}_{i}\left( \boldsymbol{r,}0 \right)\tilde{\psi}_{i}^{*}\left( \boldsymbol{r}^{'},0 \right) \right] \#\left( S14 \right) \end{aligned}$$

where the subscript *s* stands for “single-particle”. For periodic systems, the TDM can be reformulated to reflect the underlying lattice structure:

$$\begin{aligned} \Gamma_{s}\left( \boldsymbol{r},\boldsymbol{r}^{'},t \right)=\sum_{v,\boldsymbol{k}} \left[ \tilde{\psi}_{v\boldsymbol{k}}\left( \boldsymbol{x},t \right)\tilde{\psi}_{v\boldsymbol{k}}^{*}\left( \boldsymbol{x}^{'},t \right)-\tilde{\psi}_{v\boldsymbol{k}}\left( \boldsymbol{x,}0 \right)\tilde{\psi}_{v\boldsymbol{k}}^{*}\left( \boldsymbol{x}^{'},0 \right) \right]e^{i\boldsymbol{k}\cdot(\boldsymbol{R}-\boldsymbol{R}^{\boldsymbol{'}})} \#\left( S15 \right) \end{aligned}$$

Here, the $\boldsymbol{r= x+R}$, with ***x*** being the position within the unit cell and ***R*** a direct lattice vector.

Understanding the dynamics of carrier transformation between different types is crucial for gaining deeper insights into the exciton formation process and its related effects. However, in the dynamics of exciton formation, the type of carrier is not well defined. To provide a valuable physical picture, we categorize the carriers into three distinct groups: free carriers, exciton core, and full exciton. Here, free carriers denote photoexcited electron-hole pairs that have not yet developed short-range excitonic correlation and are described by delocalized interband coherence. And we operationally define an exciton core as the short-range component of the excitonic wavefunction, confined within the average exciton radius: $\psi_{ex-core}\left( \boldsymbol{r} \right)= \psi_{\mathrm{exciton}}\left( \boldsymbol{r} \right)\theta\left( \boldsymbol{r}_{\mathbf{h}}-\boldsymbol{r} \right)$.

First, we assume that the number of free carriers $n$ at any given moment is proportional to the square of the applied electric field intensity $E^{2}$, with the proportionality constant *p*_1_ = 0.043 e·Å^2^·V^-2^, and assume that *t* = 0.4 fs, the carriers are all free carriers. Next, we assume that after the laser pulse ended (*t* = 10 fs), all carriers have transitioned into full excitons. The ratio of the hole density in central region (*r* < *r*_c_ = 3.3 Å) to the hole density in periphery region (*r*_c_ < *r* < *r*_p_ = 6.7 Å) for full excitons is a constant *p*_2_ = 0.18. Using these assumptions, the proportion of each carrier type relative to the total number of carriers can be calculated by:

$$\begin{aligned} f_{\mathrm{free}}\left( t \right)=\frac{n_{\mathrm{free}}(t)}{n_{\mathrm{total}}(t)}=\frac{p_{1}{E(t)}^{2}}{n_{\mathrm{total}}(t)} \#\left( S16 \right) \end{aligned}$$

$$\begin{aligned} f_{\mathrm{full}}\left( t \right)=\frac{n_{\mathrm{full}}(t)}{n_{\mathrm{total}}(t)}=p_{2}\frac{\int_{r_{c}}^{r_{p}} {d\boldsymbol{r}_{\mathbf{h}}|\Gamma_{s}\left( \boldsymbol{r}_{\mathbf{e}},\boldsymbol{r}_{\mathbf{h}},t \right)|}^{2}}{\int_{0}^{r_{c}} {d\boldsymbol{r}_{\mathbf{h}}|\Gamma_{s}\left( \boldsymbol{r}_{\mathbf{e}},\boldsymbol{r}_{\mathbf{h}},t \right)|}^{2}}-(\frac{r_{p}^{2}}{r_{c}^{2}}-1)f_{\mathrm{free}}\left( t \right) \#\left( S17 \right) \end{aligned}$$

$$\begin{aligned} f_{ex-core}\left( t \right)=\frac{n_{\mathrm{core}}(t)}{n_{\mathrm{total}}(t)}=1-f_{\mathrm{free}}\left( t \right)-f_{\mathrm{full}}\left( t \right) \#\left( S18 \right) \end{aligned}$$

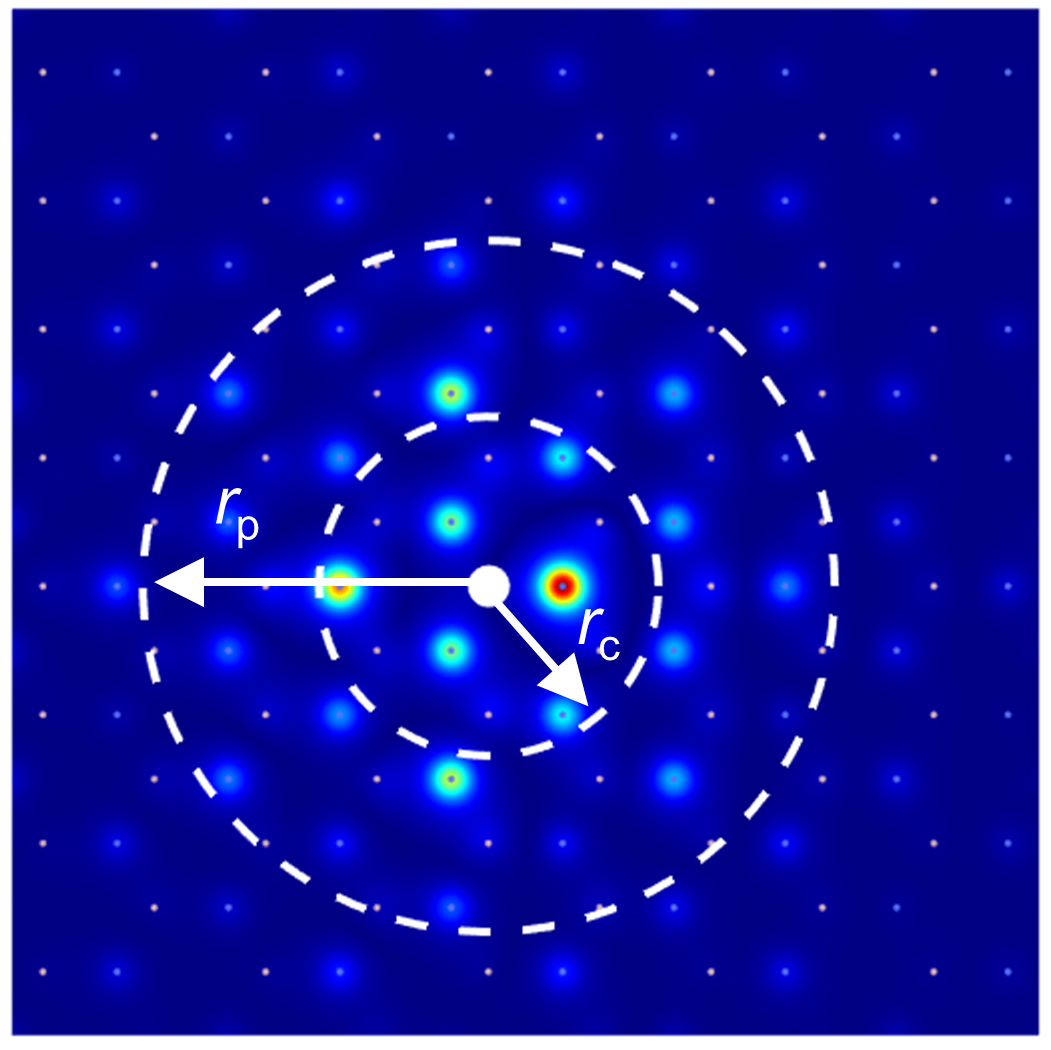


**Fig. S1 Schematic diagram illustrating the spatial distribution of carriers during the exciton formation process.** The central region (radius *r* < *r*_c_ = 3.3 Å) represents the exciton core, while the region (*r* < *r*_p_ = 6.7 Å) represents the full exciton. Here, $r_{e}$ is fixed at the position of a particular boron atom, marked with a white dot.

**Note S2. Parameter setting of the** **range-separation hybrid functional**

The RSH functional has three tunable parameters: α, β and γ. From the formula for the Coulomb interaction:

$$\begin{aligned} \frac{1}{r}=\frac{1-\left[ \alpha+\beta\mathrm{erf} \left( \gamma r \right) \right]}{r}+\frac{\alpha+\beta\mathrm{erf} \left( \gamma r \right)}{r} \#\left( S19 \right) \end{aligned}$$

and the formula for the exchange-correlation energy:

$$\begin{aligned} \begin{aligned} E_{\mathrm{XC}}=&\alpha E_{\mathrm{HFX}}^{\mathrm{SR}}+\left( 1-\alpha\right)E_{\mathrm{LDAX}}^{\mathrm{SR}}+\left( \alpha+\beta\right)E_{\mathrm{HFX}}^{\mathrm{LR}} \\ +&\left[ 1-\left( \alpha+\beta\right) \right]E_{\mathrm{LDAX}}^{\mathrm{LR}}+E_{\mathrm{LDAC}} \end{aligned} \#\left( S20 \right) \end{aligned}$$

The parameters are determined through a three-step procedure. (i) First, to ensure the correct asymptotic behavior of the exchange interaction, we impose the condition $\alpha+\beta=1$. (ii) Second, the parameters α and γ are further constrained along a one-dimensional curve by fitting to quasiparticle band gaps obtained from first-principles GW calculations (Fig. S2A). (iii) Finally, we set $\alpha=0$, based on the physical consideration that short-range local-field effects in two-dimensional insulators are adequately captured by standard local functionals such as LDA or PBE. This systematic protocol uniquely specifies all parameters, yielding a functional that accurately incorporates both short-range local-field effects and long-range exchange interactions.


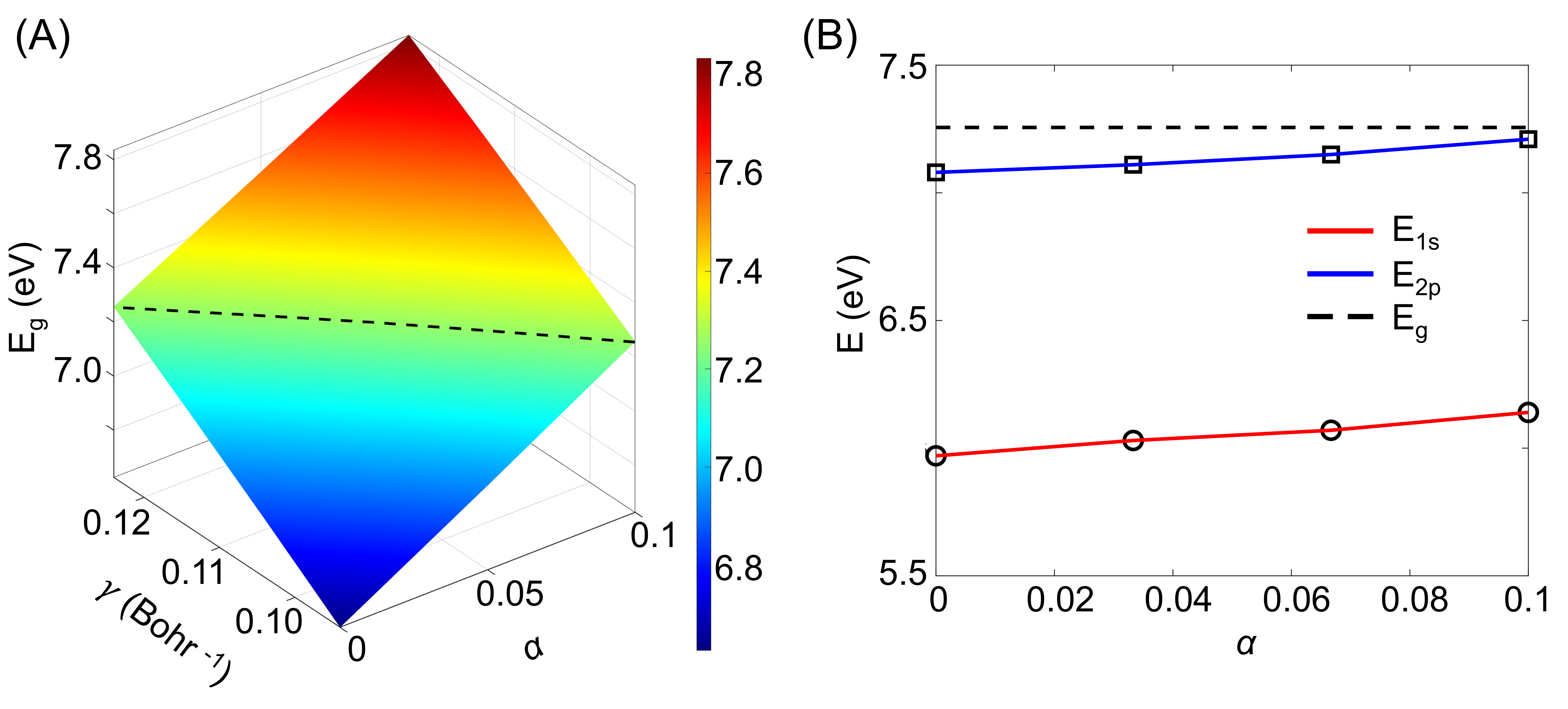


**Fig. S2 Influence of RSH parameters on the direct band gap and exciton energies.**

(A) Under the constraint $\alpha+\beta=\varepsilon_{0}^{-1}$, the direct band gap of monolayer h-BN varies with the parameters α and γ. The black dashed line denotes the parameter values that yield a direct band gap consistent with the prediction from G_0_W_0_ (B) Exciton energies of the 1S and 2P states calculated for parameter sets along the black dashed line in panel (A). The direct band gap is indicated by the dashed line for reference.

To demonstrate the generalizability of our parameterization scheme, we applied the exact same parameter-selection procedure to monolayer MoS_2_ without any re-tuning. By setting α=0, β=1, and γ=0.032 Bohr^−1^ was uniquely determined by the GW bandgap (2.50 eV). As shown in Fig. S3, our TD-RSH calculation predicts the A and B exciton peaks at 2.03 eV and 2.17 eV. This result reproduces the key features of the MoS₂ absorption spectrum, in good agreement with experimental values (approx. 1.90 eV for A and 2.04 eV for B). Therefore, this validation strongly supports that our parameterization is physically sound and transferable, rather than being fitted to a specific case.


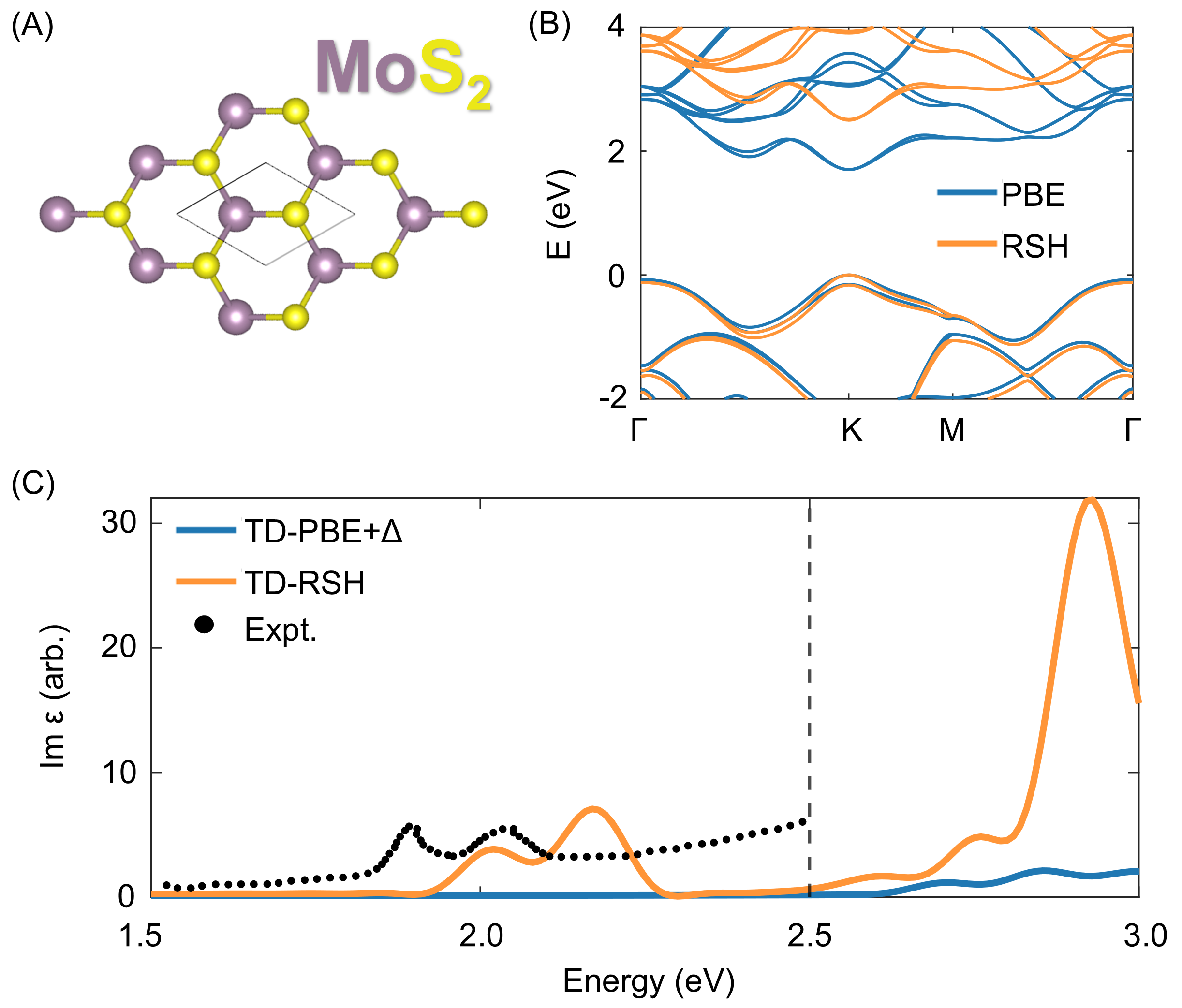


**Fig. S3. The band structure and the imaginary part of dielectric function of monolayer MoS_2_.** (A) Atomic configuration of monolayer MoS_2_. (B) The band structure of monolayer MoS_2_ calculated using DFT with the PBE functional (blue), DFT with the RSH functional (yellow). (C) The imaginary part of dielectric function of monolayer h-BN calculated using TD-PBE plus a scissor correction Δ=0.96 eV (blue) and TD-RSH (yellow). The experimental result (black dot) is shown for comparison.

The conclusions presented in the main text rely on a specific set of parameters for the functional. To ensure these findings are not an artifact of this particular choice, we investigated the sensitivity of the results to parameter variations.

**1.The effect of the parameters on the absorption spectrum**

Figure S4A shows the imaginary part of the dielectric function for varying α (0 to 0.2), with γ decreasing accordingly (0.124 to 0.059) while maintaining fixed constraints (i) and (ii). As α increases, short-range exchange strengthens at the expense of long-range interactions, reducing exciton binding energy (1.29 eV to 0.9 eV) and blue shifting the 1S and 2P peaks. However, the 1S peak intensity is largely unaffected, and the 2S peak only gradually weakens. Quantitatively, reducing γ 25% lowers the 1S binding energy by 14%, while a 52% reduction results in a 30% decrease.

**2.The effect of the parameters on the exciton dynamics and phase oscillations**

Furthermore, we investigated the influence of these parameters on the exciton dynamics. We performed simulations for three distinct parameter sets: α=0, γ=0.124 Bohr^−1^; α=0.1, γ=0.094 Bohr^−1^; and α=0.2, γ=0.059 Bohr^−1^. To ensure the results are comparable, we used an identical excitation pulse centered at 5.9 eV with an energy broadening of approximately 2 eV for all simulations. This pulse possesses a sufficient energy bandwidth to excite the 1S exciton across the different parameter sets.

Figure S4C shows that in all three cases, the exciton radius undergoes a rapid decrease within the initial 2.5 fs, followed by a gradual increase toward a stable value. This consistency demonstrates that our qualitative conclusions regarding the exciton dynamics are robust and not an artifact of our specific parameterization. Moreover, the quantitative results align with physical intuition. The stabilized exciton radii systematically increase (3.25 Å, 3.37 Å, and 3.52 Å) as the corresponding exciton binding energies decrease (1.29 eV, 1.11 eV, and 0.91 eV).

Furthermore, we analyzed the time evolution of state occupations to probe the robustness of the phase oscillations. Figure S4D plots electrons occupation over time for the case of α=0.1, γ=0.094 Bohr^−1^. The coherence-driven oscillations remain a prominent feature. However, compared to the reference case (gray line), the oscillation period slightly increases from 3.76 fs to 3.96 fs. This lengthening of the period is physically consistent with the reduced exciton binding energy, which leads to a smaller energy gap between the 1S and 2P excitonic states.

In summary, these results demonstrate that our conclusions on exciton dynamics and phase oscillations are robust and reflect the intrinsic physical behavior of the system, rather than artifacts of parameter tuning.


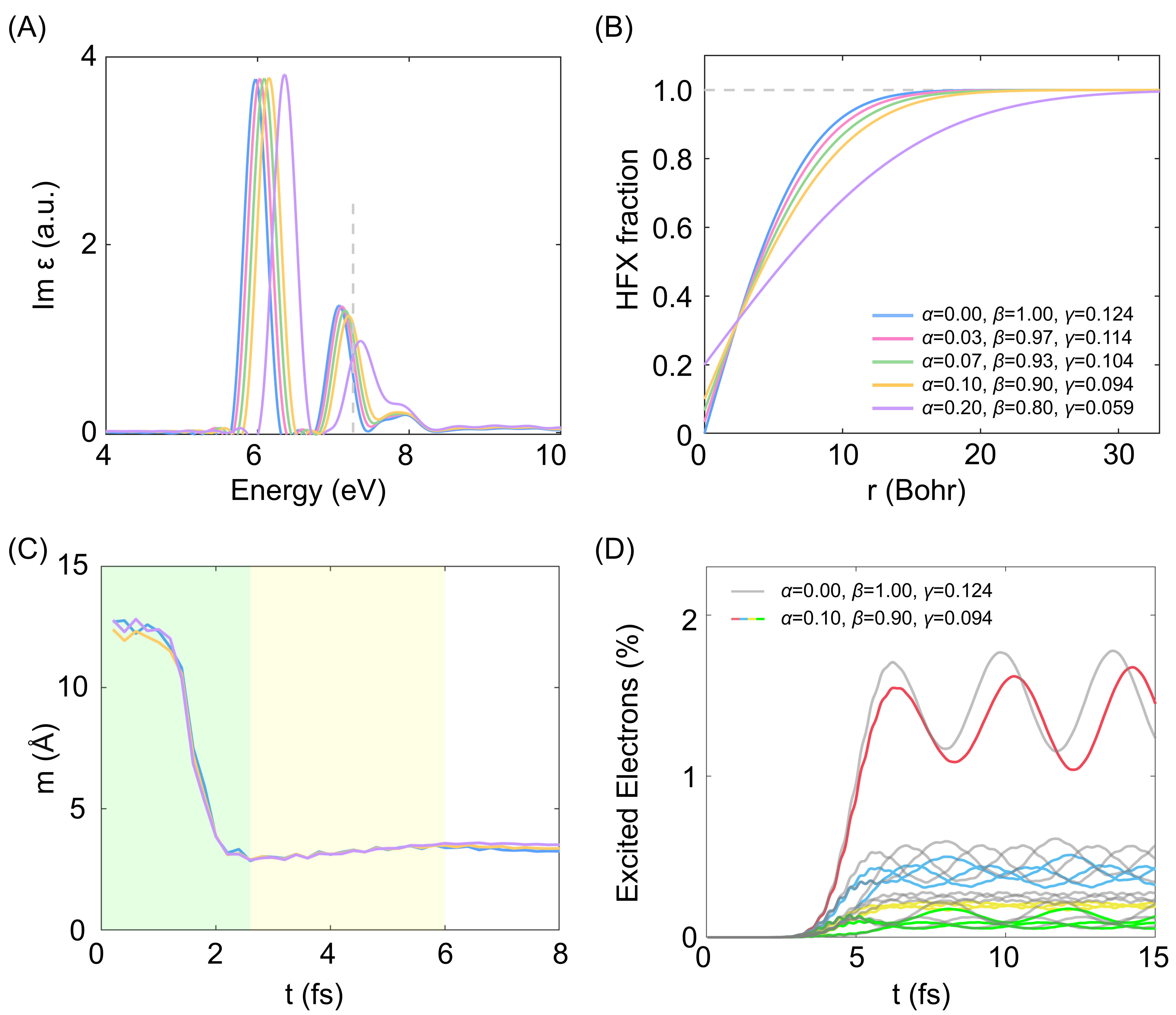


**Fig. S4 Sensitivity analysis of the parameter dependence in the TD-RSH framework.** (A) Imaginary part of the dielectric function obtained with different parameter sets. (B) Fraction of HFX as a function of inter-electron separation r, with α set to 0, 0.03, 0.07, 0.10, and 0.20 shown in blue, red, green, yellow, and purple, respectively. (C) Time evolution of the exciton radius for three representative parameter choices. (D) Time evolution of the excited electron population for α=0.1, γ=0.094 Bohr^−1^, with the gray curve denoting the reference case (α=0.0, γ=0.124 Bohr^−1^).

**Note S3.** **Dependence of Exciton Formation Dynamics on Driving Conditions**

In this note, we examine the robustness of the three-step exciton formation mechanism and the associated few-femtosecond timescale against variations in the driving laser field, focusing on both pump intensity and pulse duration.

**Effect of Pump Intensity.** To assess whether the exciton formation dynamics reported in the main text depend on the relatively strong driving field, we performed an additional TD-RSH simulation using a resonant laser pulse with a substantially reduced pump intensity of 0.56 GW·cm^-2^, corresponding to 1/16 of the intensity used in the main text. All other pulse parameters, including central frequency and temporal envelope, were kept unchanged.

Fig. S5A compares the time evolution of the average electron-hole separation *m(t)* for the strong and weak field cases. In both cases, *m* exhibits a rapid initial decrease from values exceeding 10 Å to approximately 3 Å within the first few femtoseconds, followed by a gradually increases to around 3.3 Å at later times. Consistent with this behavior, snapshots of the transition density matrix shown in Figs. S5B-D demonstrate that the same three-step exciton formation mechanism remains clearly identifiable in the weak field regime.

Quantitatively, the time at which *m(t)* reaches its minimum is modestly delayed under weaker excitation, increasing by approximately 0.5 fs compared to the strong-field case. As shown in Fig. S4, this delay arises primarily from a slower localization of the hole density around nearest-neighbor nitrogen atoms, corresponding to a slower conversion of free carriers into exciton cores. By contrast, once exciton cores are formed, their subsequent evolution toward spatially extended 1S excitons shows no pronounced sensitivity to the pump intensity within the temporal resolution of our simulations.

This trend is physically intuitive. The initial buildup of the exciton core is driven by field-induced interband coherence, which characteristic timescale is governed by an effective Rabi frequency proportional to the pulse amplitude. Reducing the field strength therefore slows the initial localization process, whereas the subsequent evolution, dominated primarily by Coulomb correlations rather than direct field coupling, exhibits a much weaker dependence on the pump intensity under the conditions explored here.

These results demonstrate that the three-step exciton formation mechanism is not dependent on strong excitation conditions, and persist in the weak field regime.


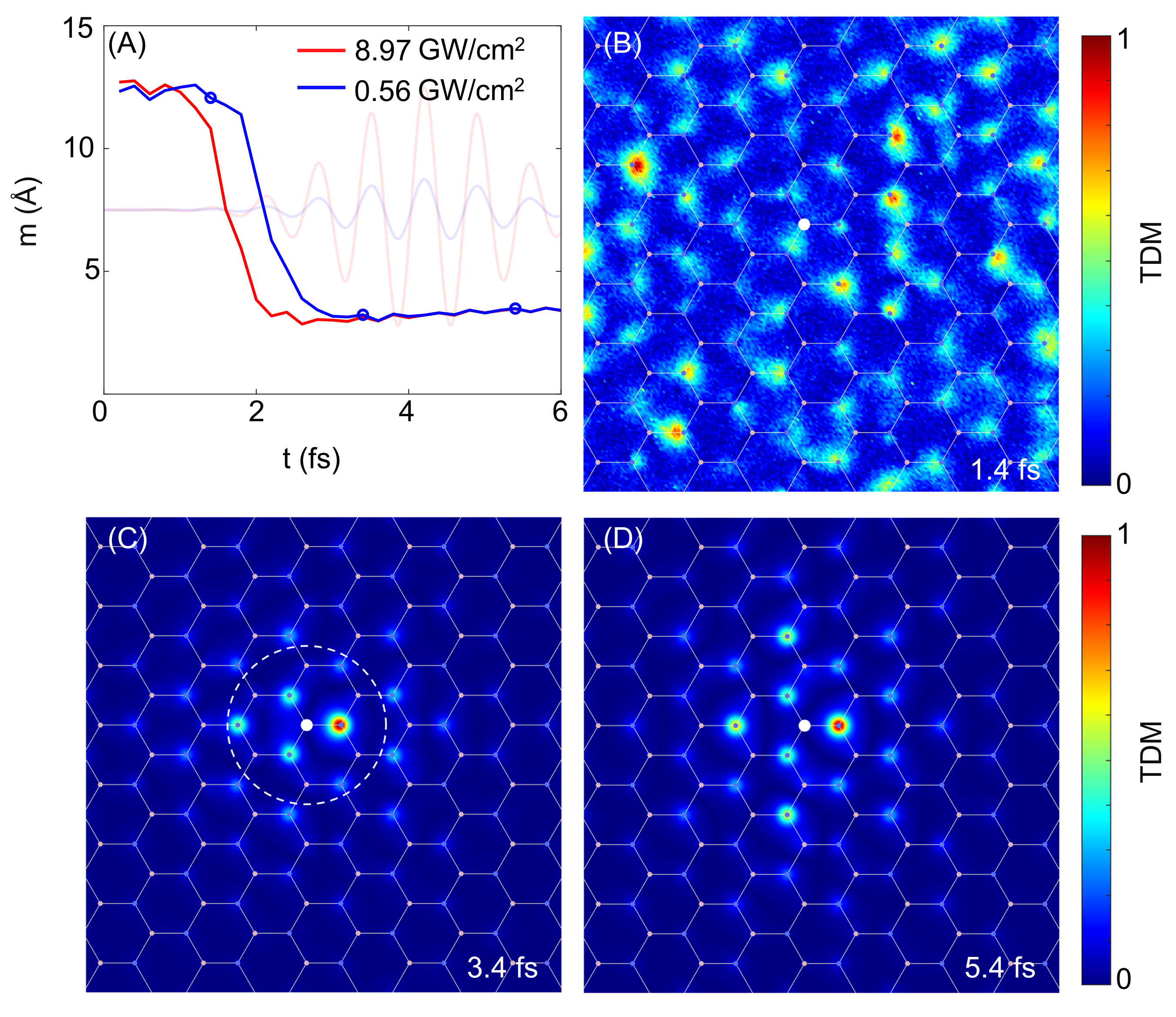


**Fig. S5 Dynamics of exciton formation in monolayer h-BN under weak pump excitation.** (A) Time evolution of the average electron-hole distance (*m*) for pump intensities of 8.97 GW·cm^-2^ (red) and 0.56 GW·cm^-2^ (blue). The laser pulse envelopes are represented by red and blue transparent solid lines. (B)-(D) The snapshot of TDM $|\Gamma_{s}\left( r_{e},r_{h} \right)|$ in real space at the times indicated by blue markers in panel (A). The exciton cores are indicated by a white dotted circle in panel (C). In each snapshot, the real-space distribution is normalized to its respective maximum intensity.

**Effect of Pulse Duration.** To address whether the ∼2.5 fs exciton formation timescale reported in the main text is limited by the duration of the ∼10 fs laser pulse, we performed an additional simulation using an ultrashort resonant pulse with a duration of approximately 1.5 fs. While such a pulse provides enhanced temporal resolution, it is accompanied by a wide energy broadening (Fig.S6B).

As shown in Fig. S6A, owing to the significantly shorter rise time, the ultrashort pulse reaches its peak electric-field strength of 0.02 V Å^-1^ in about 0.75 fs, substantially earlier than the 3.5 fs required for the ∼10 fs pulse (used in the main text) to reach a comparable field strength. This rapid field rise enhances the instantaneous effective Rabi frequency, accelerating the buildup of interband coherence and advancing the exciton core formation time. Consequently, the average electron-hole separation *m(t)* rapidly decreases from values exceeding 10 Å to approximately 3 Å within about 0.8 fs.

However, in contrast to the ∼10 fs pulse case, the average electron-hole separation increases rapidly to values exceeding 10 Å after the pulse has ended. As illustrated in Figs. S6C-L, this behavior originates from coherent coupling between excitonic states and continuum carrier states, which is induced by the large energy bandwidth of the ultrashort pulse that simultaneously excites the 1S exciton, higher-lying excitonic states, and unbound electron-hole pairs.

Under these conditions, a clean identification of a sequential three-step formation pathway becomes significantly more challenging, not because the mechanism is absent, but because the dynamics are dominated by multi-state coherence rather than by the selective buildup of a single excitonic state. This behavior reflects an intrinsic trade-off between temporal and energy resolution: while ultrashort pulses maximize time resolution, their broad bandwidth limits their suitability for selectively tracking the formation of a specific excitonic state under resonant excitation.

In this context, the ∼10 fs Gaussian pulse employed in the main text represents a balanced compromise, providing sufficient spectral selectivity to isolate the 1S exciton while still allowing access to sub-10-fs electronic dynamics relevant to exciton formation.

Taken together, these additional simulations demonstrate that the three-step exciton formation mechanism identified in monolayer h-BN is robust across a broad range of driving conditions. While the precise numerical value of the exciton formation time is not universal and exhibits a moderate dependence on pump intensity and pulse shape, the overall dynamics consistently reside within the few-femtosecond regime. Variations in the external field primarily affect the early-stage localization from free carriers to exciton cores, whereas the subsequent evolution toward fully formed excitons proceeds on comparable timescales.


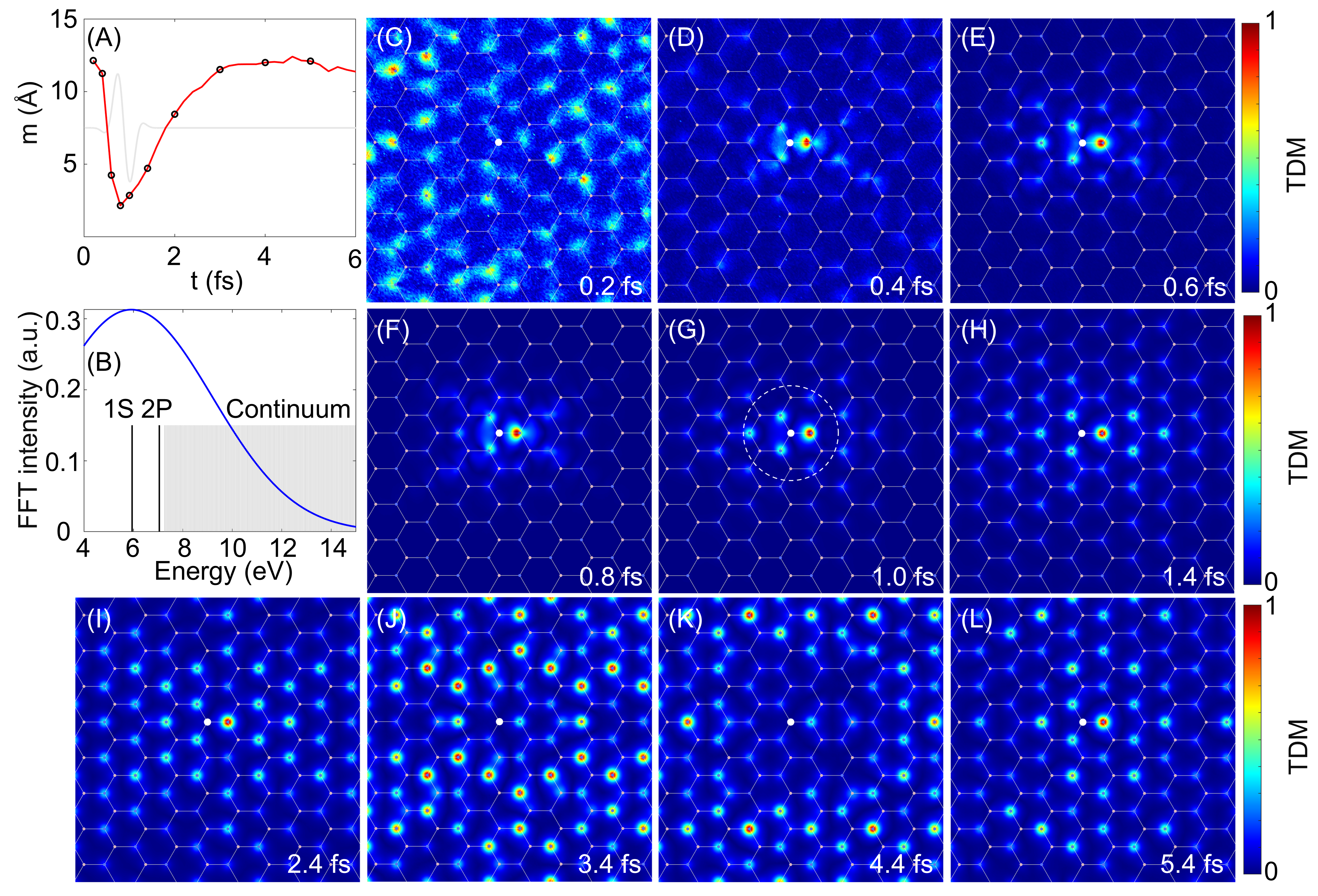


**Fig. S6 Dynamics of exciton formation in monolayer h-BN under ultrashort resonant pulse.** (A) Time evolution of the average electron-hole distance (*m*) for pump intensities of 8.97 GW·cm^-2^. The ultrashort laser pulse envelope is depicted by the gray solid line. (B) Fourier spectrum of the ultrashort laser pulse, illustrating its energy broadening. The vertical black lines indicate the energies of the 1S and 2P excitonic states, while the shaded region denotes the continuum excitation range. (C)-(L) The snapshot of TDM $|\Gamma_{s}\left( r_{e},r_{h} \right)|$ in real space at the times indicated by black markers in panel (A). The exciton cores are indicated by a white dotted circle in panel (G). In each snapshot, the real-space distribution is normalized to its respective maximum intensity.

**Note S4. Universality of the Exciton Dynamics in Wide-Gap 2D Insulators**

To examine the generality of the proposed mechanisms in wide-gap 2D insulators, we performed additional first-principles calculations on monolayer h-AlN.

**Electronic properties.** Following the parameter selection rules, we set the RSH parameters to α=0, β=1, and γ=0.081 Bohr^−1^. Unlike h-BN, h-AlN exhibits an indirect band gap of 5.24 eV (Fig. S7A). Its lowest direct transition occurs at the Γ point, with a gap of 6.02 eV. The absorption spectrum calculated using the TD-RSH method shows that the first absorption peak appears at 4.80 eV, which can be attributed to the 1S exciton at the Γ point with a binding energy of 1.22 eV (Fig. S7B). The distinct electronic structure of h-AlN makes it an excellent test case to demonstrate the universality of the proposed mechanisms.

**Exciton formation pathway.** We tracked the real-time evolution of the exciton radius following resonant excitation. As shown in Fig. S7C, the dynamics in h-AlN are strikingly similar to those in h-BN, displaying the same characteristic three-stage evolution: (i) an initial rapid contraction of the exciton radius (from >14 Å to 6 Å in ~4 fs), (ii) a subsequent partial expansion (to ~7.5 Å over ~2 fs), and (iii) final stabilization. These results indicate that h-AlN and h-BN share the same exciton formation pathway: the rapid formation of an exciton core part followed by the gradual establishment of its peripheral structure.

**Phase-sensitive dynamics.** Simulations (Fig. S7D) reveal clear oscillations in carrier occupations at different k-points, with a period of 3.36 fs that matches the 1.23 eV energy splitting between the 1S and 2P excitonic states. This demonstrates the universality of exciton quantum-beat behavior. Importantly, these oscillations exhibit distinct phases at different k-points, confirming that phase-dependent dynamics are not limited to h-BN but are a general feature of wide-gap 2D insulators.

In summary, our additional calculations on monolayer h-AlN demonstrate that both the three-stage exciton formation process and the phase-sensitive dynamics are robust and universal phenomena in wide-gap 2D insulators.


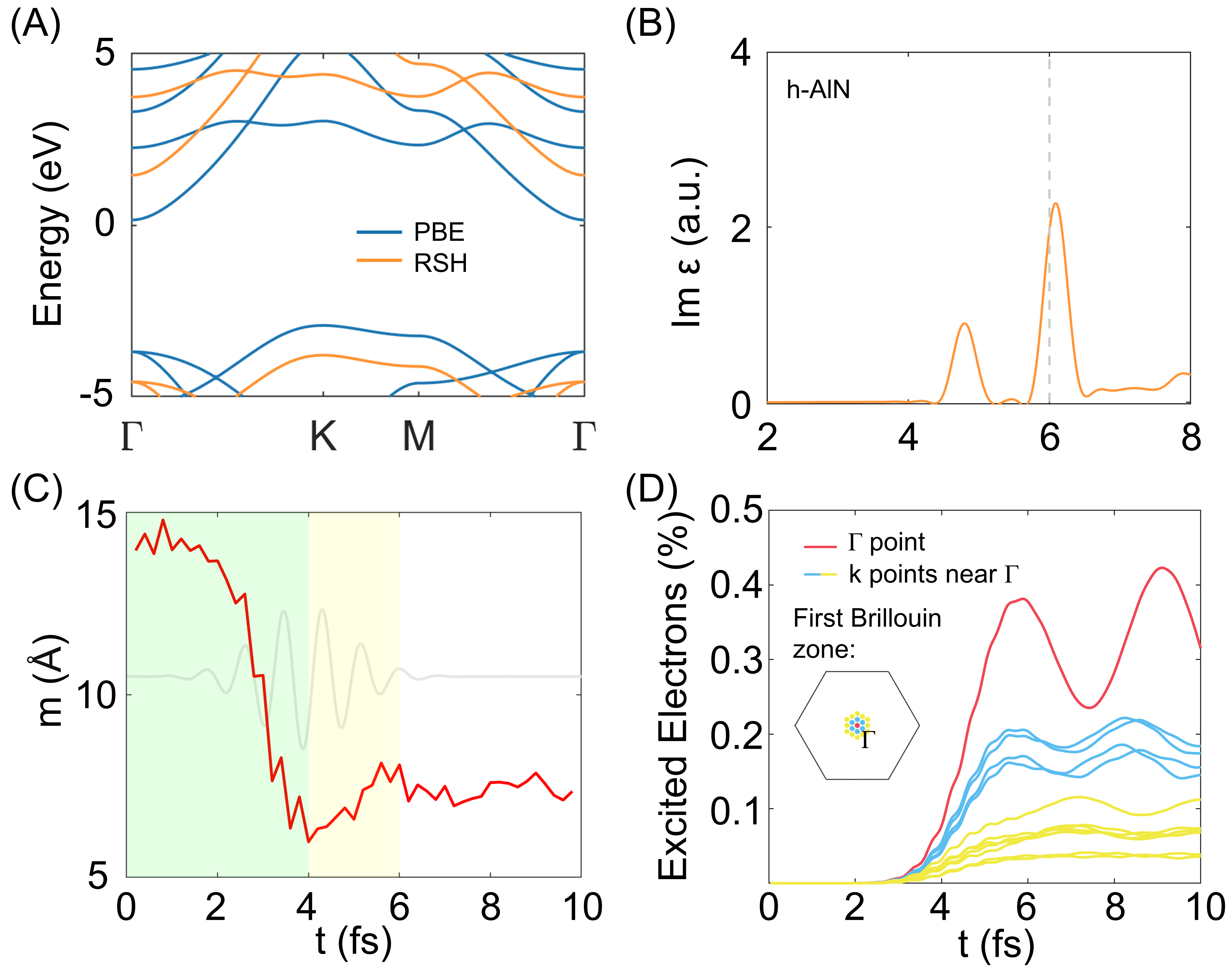


**Fig. S7 The electronic structure and exciton dynamics characteristics of monolayer h-AlN.** (A) The band structure of monolayer h-AlN calculated using DFT with the PBE functional (blue) and DFT with the RSH functional (orange). (B) The imaginary part of dielectric function of monolayer h-AlN calculated using TD-RSH (orange). Gray dashed line represents the direct bandgap. (C) Time evolution of the average electron-hole distance m. The laser pulse envelope is depicted by the gray solid line. The green and yellow shaded regions indicate stages of rapid decrease and gradual increase in electron-hole distance, respectively (D) Time evolution of the excited electron population at different k points. Inset: Position of the Γ point and the nearby k points in the Brillouin zone.


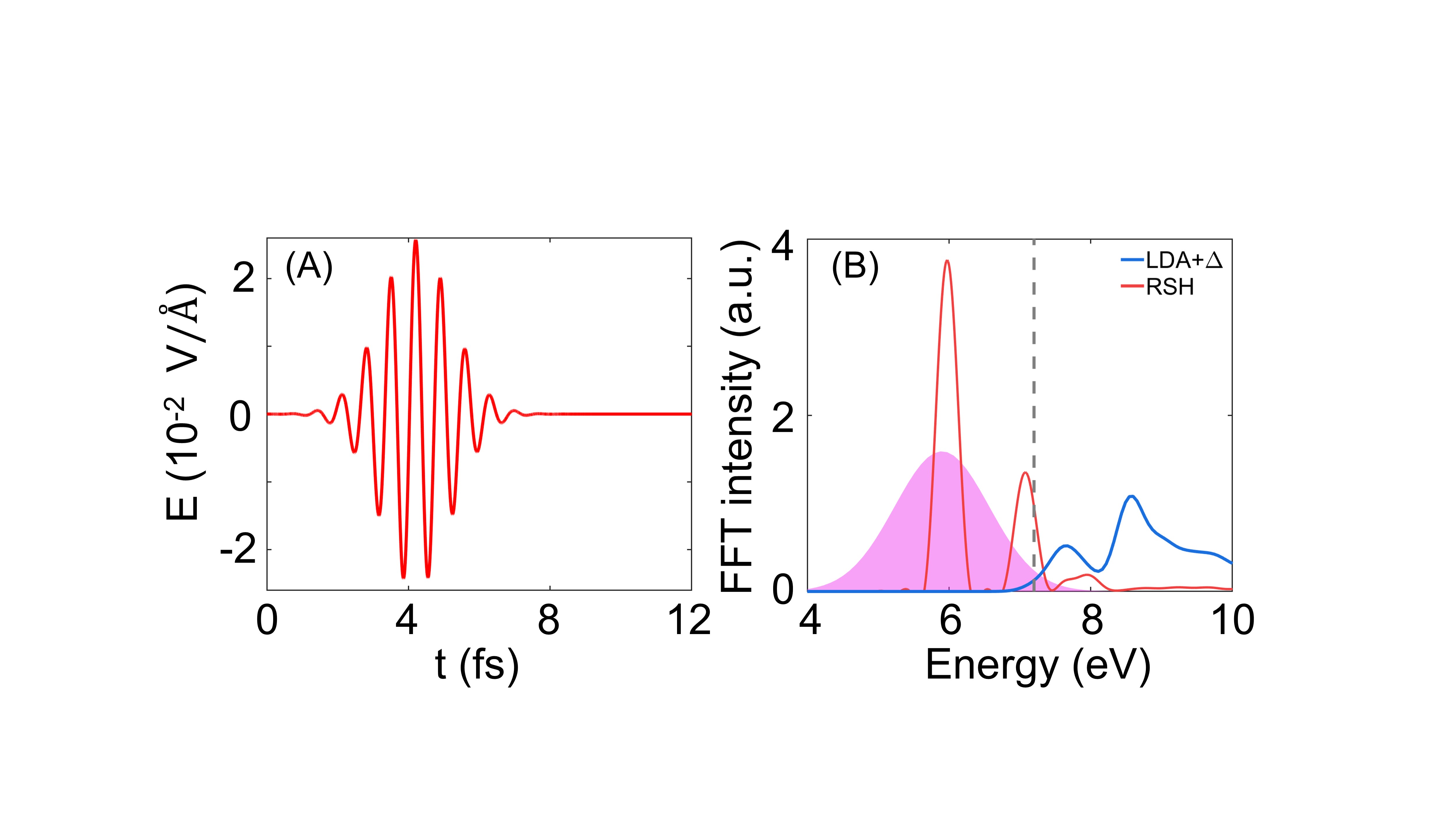


**Fig. S8 The laser pulse applied to monolayer h-BN.** (A) Waveform of the applied electric field linearly polarized along the B-N bond direction. (B) Energy broadening of the laser pulse (pink envelope). The imaginary part of the dielectric function calculated using TD-LDA and TD-RSH is also shown in the figure for reference.


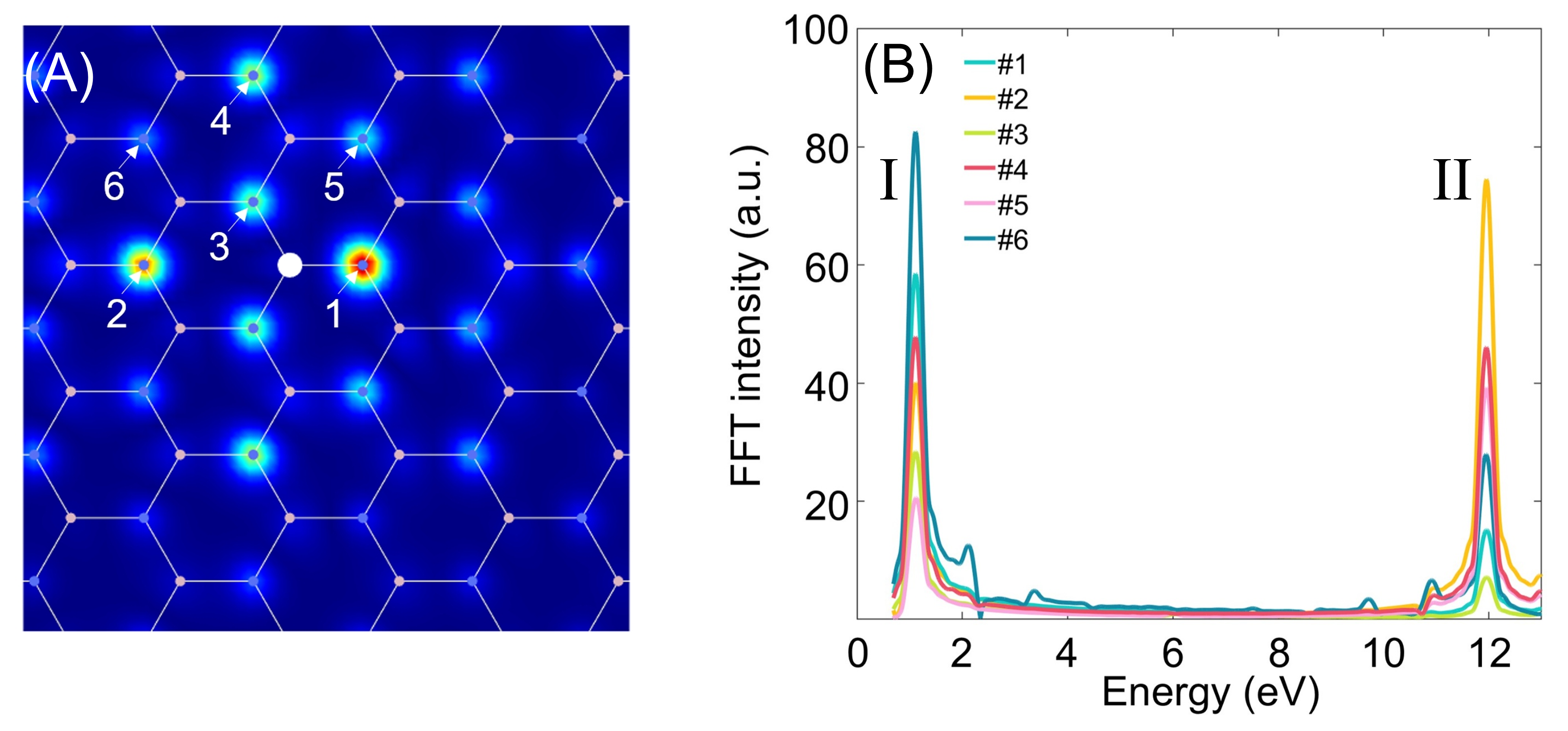


**Fig. S9 The frequency of the coherent excitonic beats.** (A) Six representative points labeled in real space. (B) Fourier transform of time evolution of the TDM at these representative points.


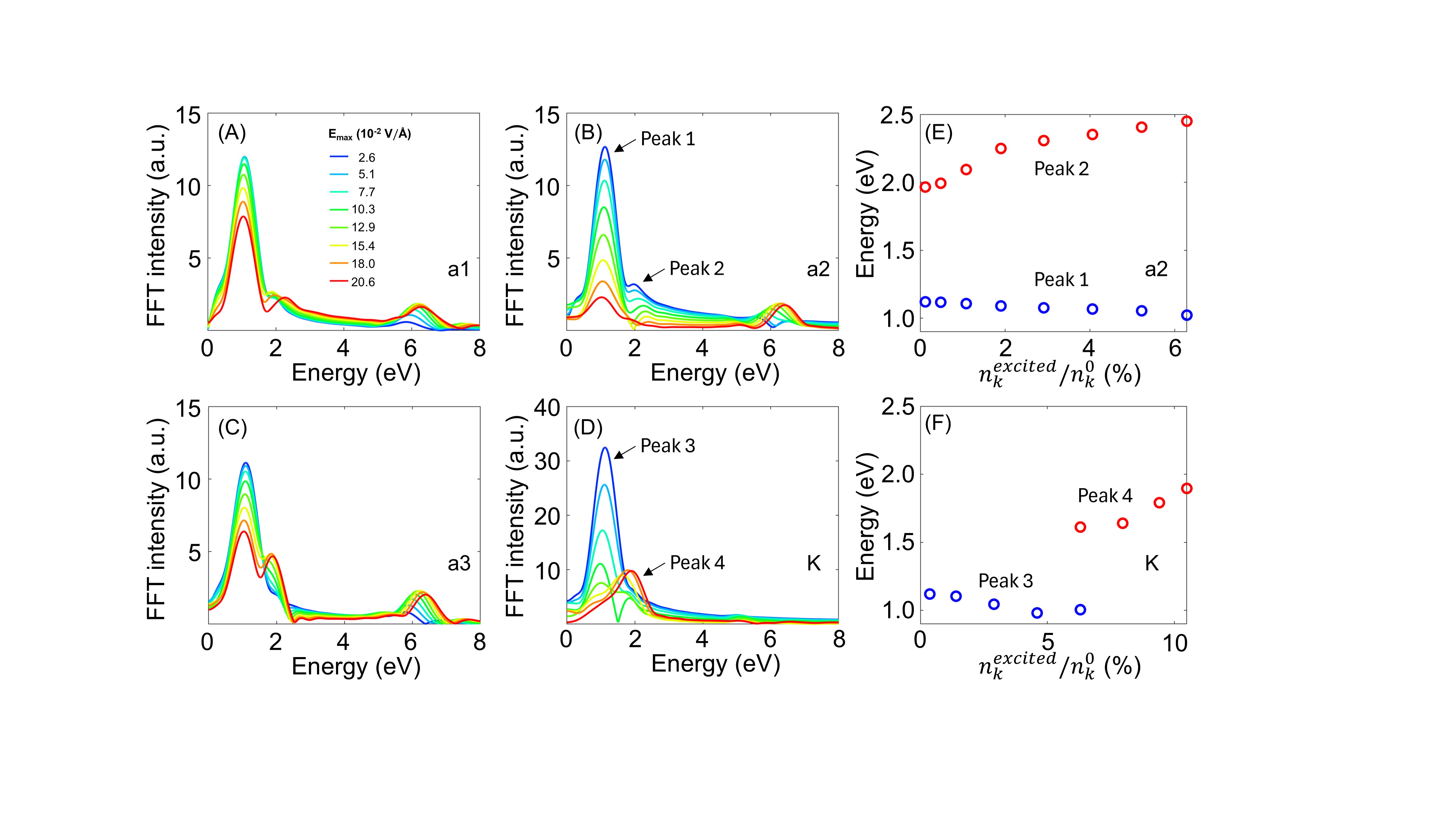


**Fig. S10 Effect of electric field intensity on coherent excitonic beats.** Fourier transform of time evolution of the excited electron population at points a1 (A), a2 (B), a3 (C), and the K point (D). The maximum electric field intensity is represented by a color gradient, with the intensity increasing from blue to red. (E) and (F) show the variation of the energies of peaks 1 and 2, and peaks 3 and 4, respectively, with the proportion of excited electrons.

**References:**

1. Runge, E. & Gross, E. K. U. Density-functional theory for time-dependent systems. *Physical Review Letters* **52**, 997-1000 (1984).
